# The developmental trajectories of working memory updating from early childhood to adolescence: A meta‐analysis

**DOI:** 10.1111/bjop.70069

**Published:** 2026-04-05

**Authors:** Ye Song, Chen Cheng

**Affiliations:** ^1^ The Hong Kong University of Science and Technology Hong Kong China

**Keywords:** childhood, developmental trajectory, meta‐analysis, task variation, working memory updating

## Abstract

Working memory updating is a crucial cognitive function for learning and academic achievement that develops significantly throughout childhood and adolescence. Despite the varieties of existing tasks to measure children's working memory updating, its overall developmental trajectory and task‐specific developmental patterns remain inadequately understood. This meta‐analysis examined 99 studies (*N* = 35,858 participants) on working memory updating performance in individuals aged 3 to 17 years, using a range of updating paradigms. Results revealed three key findings. Firstly, a significant positive developmental trend with the largest improvements was observed in early to middle childhood (ages 3–8) (*d* = 2.29). Secondly, meta‐regression analyses revealed that while both linear and quadratic models adequately described the developmental trajectory, the quadratic model provides superior fit, indicating steeper improvements in early childhood that gradually level off in adolescence. Thirdly, task‐specific analyses demonstrated distinct developmental patterns: backward recall tasks exhibited the strongest age‐related improvement (*β* = .21), whereas n‐back and selective updating tasks showed relatively flat trajectories. Together these findings suggest that working memory updating follows a curvilinear developmental progression with substantial task‐specific variations. This comprehensive analysis provides valuable insights for understanding the development of working memory updating and practical implications for age‐appropriate cognitive function measures.

Working memory (WM) is a cognitive system responsible for the temporary storage and manipulation of information essential for complex tasks such as learning, reasoning and comprehension (Baddeley, 2012). The capacity of WM is a significant predictor of academic achievements and is consistently linked to reading and mathematical abilities (Ashcraft & Kirk, 2001; Gathercole et al., 2016). Beyond educational settings, WM research has clinical applications including early detection of Alzheimer's disease (Toepper et al., 2008) and human–computer interaction design (Sears et al., 2007), underscoring its fundamental importance in human cognition across the lifespan.

A key component of WM is updating, as identified by Miyake et al. (2000) as one of the core executive functions. Updating refers to the dynamic process of retrieving and modifying stored information by replacing outdated content with new, relevant data while suppressing irrelevant details (Ecker et al., 2010; Morris & Jones, 1990). This process encompasses maintaining pertinent information in an active state, monitoring the relevance of incoming stimuli and inhibiting information that is no longer needed. Unlike standard working memory tasks, updating tasks demand active manipulation and transformation of memory contents in response to changing task demands. As cognitive demands in daily life continue to evolve, the ability to effectively update information in WM has become crucial, as it underlies various cognitive domains including reasoning, reading comprehension (Palladino et al., 2001), problem‐solving (Passolunghi & Pazzaglia, 2004; Xing et al., 2019), learning and adaptation (Lechuga et al., 2016; Pappa et al., 2020) and emotional regulation (Pe et al., 2015).

## Varieties of WM updating tasks

Updating ability can be assessed through various experimental paradigms that differ in their emphasis on capacity limitations versus executive control demands. Some tasks primarily tax working memory capacity through requirements to maintain and manipulate multiple items simultaneously, while others emphasize controlled updating processes such as selective replacement or continuous monitoring. These task‐specific characteristics may influence developmental trajectories, as capacity and control processes show distinct maturational timelines (Cowan, 2001; Diamond, 2013).

To comprehensively examine WM updating development, we focused on five commonly used paradigms from the developmental literature. First, the n‐back task presents a continuous stream of stimuli where participants must indicate whether the current stimulus matches the one presented n‐positions back, requiring constant updating of memory contents as new information replaces old. Second, the keep track task involves monitoring multiple categories of information simultaneously (e.g. animals, colours) and remembering only the most recent exemplar from each category, necessitating updating of category‐specific information. Third, the running memory task presents sequences of items of unknown length, with participants required to recall only the last few items, demanding continuous updating as new items appear and older items become irrelevant. Fourth, the selective updating task explicitly instructs participants when to update specific memory positions with new information while maintaining other positions unchanged, providing direct assessment of controlled updating processes. Lastly, the backward recall task requires participants to reproduce a sequence of items in the reverse order of their presentation, like recalling a series of digits or spatial locations from last to first. This task demands active mental reorganization of already‐encoded sequential information, requiring participants to reorder item positions within working memory rather than simply retrieve them as stored. Because each item's position must be mentally reassigned during recall, backward recall engages manipulation processes that overlap conceptually with updating. Notably, backward recall tasks also impose comparatively higher working memory capacity demands than the other four paradigms, as participants must simultaneously maintain the full sequence while executing the reversal operation. Given these dual demands on both capacity and manipulation, the classification of backward recall tasks as updating measures remains a subject of debate in the field, a controversy we address in detail in the present study section.

## Literature review of WM updating in development

WM updating develops substantially from early childhood through adolescence, with refinement of underlying neurocognitive pathways and improvements in both the manipulation capacity (the ability to actively transform information in working memory) and the flexibility (the ability to shift between different mental sets or task demands) (Davidson et al., 2006; Ferguson et al., 2021). This development makes WM updating an increasingly important cognitive predictor and fundamental building blocks supporting academic achievement across domains, including reading comprehension (Ness & Meltzer‐Asscher, 2018; Zaccoletti et al., 2020) and mathematics ability (Lee & Bull, 2016; Zhang et al., 2018). Furthermore, training programmes using updating paradigms have demonstrated improvements in related cognitive abilities and academic performance (Au et al., 2016; Pappa et al., 2020). Deficits in WM updating are related to various developmental disorders, including reading difficulties (Peng et al., 2013), mathematics difficulties (Iuculano et al., 2011) and intellectual disability (Carretti et al., 2010), suggesting that understanding these cognitive processes can help support struggling populations in educational settings.

### Developmental evidence of WM updating

WM updating abilities emerge early in childhood. During the first year, infants have demonstrated rudimentary updating abilities with substantial limitations that gradually diminish with age (Cheng et al., 2019; Newcombe et al., 1999; Richardson & Kirkham, 2004). For example, primitive formats of updating processes, such as simple location changes, can be observed as early as 9–12 months of age using the A‐not‐B task (e.g. Diamond et al., 1994). However, more advanced updating abilities, involving complex manipulation and conflict resolution, emerge much later and continue to develop between 18 and 30 months of age (Cheng et al., 2019; Diamond, 2013; Ganea & Harris, 2013), marking a shift from passive to proactive manipulation and updating of information.

Later, WM updating abilities show distinct developmental patterns across childhood and adolescence, characterized by non‐linear progression and substantial individual variability (Cowan et al., 2006; Lee et al., 2013; Nelwan et al., 2022). These complex developmental trajectories occur during a crucial period for shaping learning and reasoning processes. To better understand the developmental mechanism over these periods, below, we reviewed previous work examining WM updating development from childhood through adolescence.

#### Early childhood (3–5 years)

Early childhood updating development is characterized by improvements in the ability to control WM contents and increasing strategies to handle the complexity in updating demands. Freier et al. (2021) investigated WM gating strategies in children aged 3 to 7 years using an adapted input/output gating paradigm. Findings revealed that starting from age 3, children can selectively update WM contents when the contextual cue appears early and perceptual stimuli are concrete, and that their performance showed significant improvements with age. Moreover, children's use of WM updating strategies became more flexible when they had agency over task goals rather than when goals were externally imposed.

However, these emerging abilities face significant challenges under increased demands. In one study, Cheng and Kibbe (2022) systematically examined the role of storage and updating demands in the development of visual WM updating ability in 4–7‐year‐olds. In the task, children were asked to track arrays of hidden items in WM that either remained static or swapped locations. Results revealed that 4‐year‐olds could update information in WM under minimal demands but struggled with increased updating loads. Furthermore, the study found that updating imposed a significant one‐time cost to WM performance, regardless of the number of swap movements. This suggests that the initiation of updating processes, rather than the maintenance of tracking, represents one of the primary developmental bottlenecks in WM updating, with performance improving markedly from ages 4 to 7. Similarly, 4–5‐year‐olds struggled with simultaneous maintenance and manipulation of multiple pieces of information in a ‘Magic House’ task—an adapted running memory task where children must remember the last two animals that entered a house sequentially (Panesi & Morra, 2022).

Early development of WM updating has shown significant influence in later academic outcomes. In Lee and Bull's cross‐sequential study (Lee & Bull, 2016), 673 children were tested using multiple WM measures, including a pictorial running memory task. The results showed that kindergarten‐level updating abilities predicted later mathematical achievement. This predictive relationship remained significant even after controlling for domain‐specific mathematical knowledge. Importantly, higher updating capacities at kindergarten predicted faster mathematical growth rates across all subsequent grades. The effect sizes were particularly strong for early elementary school children, suggesting a critical window during which updating abilities show a stronger impact on academic development. This critical period aligns with broader developmental patterns identified by Karr and colleagues' network analysis (Karr et al., 2022), which examined 3944 participants using a list sorting task and revealed that updating abilities in early childhood (ages 3–5) are initially intertwined with other cognitive skills but gradually become a distinct, specialized ability by school age.

#### Middle childhood (6–8 years)

Entering formal education, middle childhood marks a period of rapid advancement in updating abilities. Pelegrina et al. (2015a, 2015b) documented this progression using n‐back tasks, which require continuous updating of WM content. In the tasks, children viewed a sequence of stimuli and were asked to determine whether the current item matches the one presented ‘n’ steps back in the sequence. Results showed that while 6‐year‐olds were able to complete 1‐back trials, they struggled with 2‐back trials that demanded tracking and updating two sequential positions simultaneously. In contrast, 7–8‐year‐olds demonstrated emerging competence in 2‐back trials, indicating an expansion in updating capacity. This capacity expansion enabled more complex cognitive operations, as demonstrated by Whitely and Colozzo (2013), who found that updating abilities significantly predicted narrative skills in children from kindergarten to grade 2. Children with stronger updating skills showed better management of story elements, including tracking character actions, maintaining plot coherence and integrating new information into ongoing narratives.

This developmental progression is further supported by longitudinal evidence between ages 6–9 in a backward digit span task, where children listen to a sequence of numbers and then recall them in reverse order (Lensing & Elsner, 2018). Their findings showed different developmental trajectories between age cohorts. The younger cohort (6–7 years) had steeper growth curves than older children (8–9 years), suggesting a levelling off in WM updating development during middle childhood. Interestingly, while both cohorts showed significant age‐related improvements, inter‐individual variance in the rate of change was significant only in the older cohort, indicating a more heterogeneous developmental pattern.

Hu et al. (2023) further clarified the academic implications of these developing updating abilities through a longitudinal study of 131 Chinese students examining the cascading effects of WM updating on mathematical performance. Using both verbal and visuospatial n‐back tasks to assess updating abilities across multiple domains, they found that verbal WM updating at age 7–8 predicted basic arithmetic performance 2 years later. Similarly, visuospatial WM updating predicted the development of storage capacity in visuospatial WM, which then influenced logical–visuospatial mathematical abilities. These findings collectively suggest that middle childhood represents a critical transition period in the development of WM updating abilities, with children progressing from managing simple updating tasks to applying these skills in more complex academic and everyday contexts.

#### Late childhood (9–11 years)

Late childhood shows substantial refinement in updating capacity and integration with higher order cognitive functions, facilitated by prefrontal cortex maturation that allows for more coordinated and efficient executive processing (Zelazo & Carlson, 2012). Pelegrina et al. (2015a) and Pelegrina et al. (2015b) demonstrated that by age 9–10, children could successfully manage 2‐back tasks with relatively high accuracy, while 11‐year‐olds approached young adolescent performance levels. Additionally, Conklin et al. (2007) found that while basic maintenance abilities as measured by forward span and recognition tasks were relatively well established by age 9, more complex updating processes requiring simultaneous storage and manipulation continued developing throughout late childhood, though still not reaching adolescent levels of efficiency. This pattern suggests variation in developmental timelines resulting from the different types of updating tasks employed.

Meanwhile, the enhanced updating efficiency enables stronger connections with fluid intelligence. Belacchi et al. (2010) found that updating abilities became more predictive of complex reasoning performance in 9–11‐year‐olds compared to younger children, indicating increased specialization and systematic connection to abstract reasoning abilities. The plasticity of updating abilities during this period is highlighted by Chen et al. (2018), who used a running memory task as the target training task on 9–11‐year‐olds. Children receiving targeted updating training showed improvements in both the trained tasks and untrained measures of WM and academic performance, with training effects maintained at the 6‐month follow‐up. These training effects were particularly pronounced for those children who initially showed lower updating abilities, suggesting both an optimal developmental window for intervention and individual differences in training responsiveness.

#### Early adolescence (12–14 years)

Early adolescence is characterized by updating abilities being actively used in sophisticated, domain‐specific applications. The ongoing maturation of the prefrontal cortex supports enhanced neural development, with brain network modules becoming increasingly segregated to facilitate executive performance in complex, real‐world tasks (Baum et al., 2017; Friedman & Robbins, 2022; Zelazo & Carlson, 2012). Jolles et al. (2012) demonstrated that 12‐year‐old children showed increased frontoparietal activation following working memory training, developing more adult‐like neural patterns that suggest enhanced recruitment of mature brain circuits through practice.

This neural optimization supports enhanced academic performance. Yen et al. (2017) examined updating ability using a running span task alongside measures of number sense in junior high school students. They found that updating abilities significantly predicted mathematical achievement, even after controlling for other cognitive factors. This association was particularly strong for complex mathematical tasks which required simultaneous manipulations of multiple pieces of information, demonstrating the critical role of updating in mathematical reasoning during early adolescence. Similarly, Swanson et al. (2006) investigated different components of WM in relation to reading abilities. Their findings revealed that updating abilities became particularly important for reading comprehension tasks requiring integration of new textual information with previously read content.

Beyond academic domains, updating abilities support complex real‐world cognitive operations in early adolescents. Basso et al. (2023) examined prospective memory in 8‐ and 12‐year‐old children, finding that stronger updating abilities enable better maintenance and execution of future intentions. Children with stronger updating skills showed enhanced ability to monitor changing environmental cues while maintaining intended action.

#### Late adolescence (15–17 years)

Late adolescence marks the near‐culmination of updating development, with performance approaching adult levels while retaining subtle developmental differences. This period is marked by increased strategic control and integration within the broader executive function network, supported by more efficient modular brain connectivity (Baum et al., 2017; Karr et al., 2022; Poon, 2018).

Conklin et al. (2007) documented this near adult‐level performance, revealing that 15–17‐year‐olds achieved accuracy levels comparable to adults on backward recall tasks. However, their findings also showed that adolescents' performance remained slightly less efficient under high cognitive loads, reflecting continued dorsolateral prefrontal cortex maturation. Building on this foundation, Carriedo, Corral, Montoro, Herrero, Ballestrino, and Sebastián (2016) and Carriedo, Corral, Montoro, Herrero, and Rucián (2016) found that updating performance continued to improve significantly through late adolescence using keep track and running memory tasks. Their findings revealed that 15–17‐year‐olds showed enhanced strategic control and reduced interference effects compared to younger adolescents. The sophistication of updating processes during this period enables complex linguistic operations requiring abstract reasoning. They further found that updating abilities strongly predicted metaphor comprehension, particularly for novel metaphors requiring integration of abstract concepts. This relationship was stronger in 15–17‐year‐olds, reflecting the integration of updating abilities with advanced abstract reasoning capabilities.

## Literature gaps

While numerous studies have investigated WM updating in different age groups across development, most have focused on specific age ranges. For example, the majority of existing work has concentrated on late childhood (9–11 years), accounting for 113 of the 317 datasets (36%) in our meta‐analysis sample. Less attention has been given to early childhood and late adolescence. This imbalance has created a gap in understanding the broader developmental trajectory of WM updating from early childhood through late adolescence (Best & Miller, 2010; Voigt et al., 2014).

Without a clear developmental trajectory, identifying normative development benchmarks becomes difficult, which are essential for detecting atypical patterns in children with cognitive delays or neurodevelopmental disorders. While early childhood is recognized as an emergent period for WM updating (Karr et al., 2022), it remains unclear how early updating abilities predict later developmental outcomes. Similarly, while late adolescence is often associated with near‐adult updating performance (Conklin et al., 2007), subtle developmental differences persist (Karr et al., 2022), yet these nuances are rarely explored in relation to earlier stages.

This gap may stem from the limited number of cross‐sectional studies comparing multiple age groups across broader developmental spans and the scarcity of longitudinal research tracking children's WM updating over time. One challenge that may cause this age‐sampling limitation is the methodological inconsistency, where different updating tasks are often employed for different age ranges, making direct cross‐sectional comparisons problematic. Researchers often employ paradigms with varying cognitive demands: simplified tasks for younger children and more complex paradigms for older participants. Consequently, determining whether the observed developmental differences reflect genuine cognitive maturation or task‐specific demands becomes difficult. Moreover, many researchers employ these tasks as general measures of WM capacity rather than as specific updating assessments. This conflation is problematic because, though related, WM capacity and updating are conceptually distinct constructs (Bandettini et al., 2025; Morra et al., 2018). This practice further challenges the efforts to establish age‐appropriate normative benchmarks and prevents equitable developmental comparisons.

Different updating paradigms reveal task‐specific developmental trends that challenge unified trajectory notions. For instance, n‐back tasks show steady improvements through adolescence, while backward recall tasks demonstrate rapid early development followed by later plateaus (Pelegrina et al. (2015a) and Pelegrina et al. (2015b); Skalaban et al., 2022). Keep track and running memory tasks also show distinct patterns, possibly reflecting different updating demands and cognitive loads (Carriedo, Corral, Montoro, Herrero, Ballestrino, & Sebastián, 2016; Carriedo, Corral, Montoro, Herrero, & Rucián, 2016). These findings suggest WM updating development may not follow a singular path but instead reflect the interplay between task‐specific demands and broader cognitive changes.

## The present study

The literature reviewed above highlights two key gaps in our understanding of WM updating development. First, most existing studies focus on narrow age ranges, leaving the full developmental trajectory from early childhood through late adolescence poorly characterized. Second, the use of different task paradigms across studies makes it difficult to determine whether observed developmental differences reflect genuine cognitive maturation or task‐specific demands. In the following meta‐analysis, we aim to address these gaps by examining WM updating development across a broad age range and multiple task conditions. Specifically, we will model the developmental trajectory of WM updating performance based on results from current literature with participants aged 3 to 18 years, capturing changes across the entire developmental period. In addition, we will evaluate the task‐specific developmental pattern to examine whether different paradigms reveal distinct developmental patterns.

As described above, four of the five paradigms included in this meta‐analysis (n‐back task, keep track task, running memory task and selective updating task) represent well‐established updating measures with relatively clear cognitive interpretations. The fifth paradigm, backward recall task, warrants careful consideration, as it remains controversial in the field. Our inclusion is based on the observation that backward recall typically requires mental reorganization and reversal of already‐encoded sequences (Diamond, 2013; Li & Lewandowsky, 1995; St Clair‐Thompson, 2010), which operationally involves updating item positions in working memory. The cognitive processes underlying backward recall performance likely vary substantially. Adult participants sometimes employ sophisticated recursive strategies, such as immediately repeating the final digit, then mentally rehearsing the entire list to retrieve the penultimate item, and repeating this process recursively (Li & Lewandowsky, 1995). Such strategies genuinely involve updating item positions as new items are retrieved. However, young children typically do not employ even simple rehearsal strategies in forward recall tasks (Flavell et al., 1966), making it unlikely to use these sophisticated updating strategies in backward recall. Instead, older children may maintain a list of items and mentally ‘scan’ it in backward order—a form of manipulation that does not necessarily constitute updating in the strict sense. Young children (up to approximately 5–6 years) often struggle to reverse more than two items, which may reflect working memory capacity limitations rather than updating process deficits per se (Isaacs & Vargha‐Khadem, 1989; Cowan, 2016). Additionally, when backward recall involves spatial materials (e.g. Corsi blocks), holistic pattern‐based strategies may be employed that further complicate interpretation (Donolato et al., 2017).

Given these complexities, backward recall tasks occupy a controversial position between measures of working memory capacity and updating ability. Compared to the other four updating tasks, backward recall tasks place greater demands on working memory capacity in addition to information manipulation. As a result, many researchers employed backward recall tasks as a measure of working memory capacity that involves active manipulation. However, compared with other commonly used tasks that measure WM capacity in developmental populations (e.g. forward recall task, change detection task, object occlusion tasks), the backward recall task stands out for its unique expenditure on executive control processes, including monitoring, inhibition and cognitive flexibility. Furthermore, considering its operation in manipulating stored information by reordering the position to recall, an updating process occurs when each item's position must be switched to a new one.

Therefore, despite ongoing debates regarding whether the backward recall task should be classified as a measure of WM capacity or WM updating tasks, in the present study, we considered it important to include this task in our meta‐analysis. Accordingly, in the following results section, we conducted two sets of analyses: one including five tasks, and another excluding the backward recall task. Furthermore, by conducting sensitivity analyses both with and without the backward recall task, we were able to quantify its specific contribution to the observed developmental patterns and offer guidance for future research on task selection.

By examining experimental findings across different developmental groups using these paradigms, it allows us to evaluate how working memory updating changes over the developmental periods, which further contributes to the theoretical understanding of updating mechanisms across development.

## METHODS

The protocol for this review and meta‐analysis was pre‐registered with PROSPERO (https://www.crd.york.ac.uk/PROSPERO/view/CRD420251000885) and follows PRISMA guidelines.

### Literature search

Literature search was performed on 31 March 2025 using two databases: PubMed and PsycINFO, and related references from the retrieved studies. To ensure comprehensive coverage, we searched for ‘working memory’ in combination with either ‘updating’ or ‘manipulation’, as these terms are often used interchangeably. In addition, to identify studies focusing on child populations, we used the developmental terms ‘child’, ‘children’, ‘development’ and ‘age’. The complete search string was: (working memory) AND (manipulation OR updating) AND (child OR children OR development OR age), and yielded 1696 results on PubMed, 1035 results from PsycINFO and 68 studies from references, giving a total of 2799 studies.

An initial screening of titles and abstracts was conducted independently by two reviewers. Studies were selected for full‐text review if they were empirical studies investigating WM ability and potentially included child participants. Non‐peer‐reviewed studies or studies written in a non‐English language were excluded. After removing duplicates, full‐text screening used eligibility criteria.

### Eligibility criteria

The inclusion criteria for the final analysis were as follows: (a) peer‐reviewed empirical studies only, (b) healthy, typically developing participants (for studies involving clinical patients, only data from healthy control participants were included), (c) participants under 18 years (mixed age studies included only child data if reported separately) and (*d*) at least one WM updating task with behavioural performance data.

### Data extraction

Data extraction was performed independently by two reviewers, with disagreements resolved through discussion or consultation with a third reviewer. Performance data (means, standard deviations) and task descriptions were recorded across groups and conditions.

WM updating tasks identified across the reviewed studies were categorized into five types based on their distinct cognitive features and demands: (1) backward recall tasks that required active transformation or rearrangement of information (Li & Lewandowsky, 1995); (2) n‐back tasks, which included 1‐back, 2‐back and higher n‐back variations, and required monitoring and matching items across a specific lag in sequence (Kirchner, 1958); (3) keep track tasks that demanded recalling items from specific categories within a presented list (Yntema & Trask, 1963); (4) running memory tasks that involved continuous monitoring and updating of the most recent items in a sequence (Miyake et al., 2000); finally, (5) selective updating tasks, which examined the ability to replace specific items in memory while maintaining others unchanged (Ecker et al., 2010). Unlike the first four categories, which represent standardized tasks with well‐established protocols, selective updating tasks encompassed various paradigms that operationally met the definitional criteria of requiring selective modification of memory content (Artuso & Palladino, 2016; Cheng & Kibbe, 2022).

The analysis comprised two parts. First, all performance data were logit‐transformed using $\text{logit}\left(p\right)=\ln\left(\frac{p}{1-p}\right)$, where *p* represents the proportion of correct responses, to stabilize variance (Lipsey & Wilson, 2001; Nyaga et al., 2014). The variance was calculated using $\mathrm{Var}=\frac{1}{\mathrm{np}\left(1-p\right)}$.

Second, for studies containing participant data from multiple age groups, performance was compared between age groups within each study. The Cohen's *d* effect sizes were calculated to analyse performance differences between groups. Participants were categorized into five age groups: 3–5, 6–8, 9–11, 12–14 and 15–17 years old.

Cohen's *d* effect size (Cohen, 1988) was calculated as:

(1) $${\text{Cohen}}^{'}s\;d=\frac{M_{1}-M_{2}}{{\mathrm{sd}}_{\text{pooled}}}$$

where *M*_*1*_ and *M*_*2*_ represent the mean performance values for the compared groups, and sd_pooled_ is the pooled standard deviation calculated as:

(2) $$\begin{array}{c} {\mathrm{sd}}_{\text{pooled}}=\sqrt{\frac{\left(n_{1}-1\right){\mathrm{sd}}_{1}^{2}+\left(n_{2}-1\right){\mathrm{sd}}_{2}^{2}}{n_{1}+n_{2}-2}} \end{array}$$

where n1 and n2 represent the sample sizes of each group, and sd_1_ and sd_2_ represent their respective standard deviations.

The standard error of Cohen's *d* (SEd) for the meta‐analysis was calculated using:

(3) $$\begin{array}{c} {\mathrm{SE}}_{d}=\sqrt{\frac{n_{1}+n_{2}}{n_{1}n_{2}}+\frac{d^{2}}{2\left(n_{1}+n_{2}-2\right)}} \end{array}$$

### Quality assessment

A quality evaluation matrix was developed to assess the included studies based on two key criteria: the comprehensiveness of participant characteristics reporting and the methodological rigour (see Supplementary Material for detailed evaluation criteria). Given this study's focus on developmental trends in WM updating ability, particular emphasis was placed on age range to avoid overlapping age groups that could confound results. Additionally, the methodological quality assessment was particularly important given the wide variety of updating measures utilized across studies. This methodological evaluation focused on the justification of parameters, clarity of measures and standardization of instructions and administration procedures to ensure reliable comparisons across studies.

### Statistical analysis

Analyses were conducted using R (Version 4.4.1; R Core Team, 2024) with the metafor package (Version 4.6.0; Viechtbauer, 2010). Given the expected heterogeneity across studies and our interest in developmental moderators, we employed a mixed‐effects framework for the main analysis, which extends the random‐effects model by incorporating moderator variables. This approach allows us to examine how study characteristics influence effect sizes and explain heterogeneity between studies (Dettori et al., 2022; Hedges, 1992).

To comprehensively characterize the developmental trajectory of WM updating, we implemented a hierarchical analytical framework that progressed from broad developmental patterns to specific task‐dependent effects. The primary analysis employed a mixed‐effects meta‐regression model with age as a continuous moderator, using logit‐transformed effect sizes to address distributional assumptions and stabilize variance at the extremes.

To validate these findings and address potential limitations of cross‐sectional comparisons, we conducted targeted subgroup analyses focusing on studies that assessed multiple age groups within the same experimental protocol. By calculating within‐study effect sizes between age groups, this approach controlled for methodological variations that might otherwise confound developmental interpretations. Building on these developmental findings, we conducted additional analyses to identify the best‐fitting model for explaining changes in effect sizes across development.

Finally, to examine whether developmental trajectories varied as a function of task demands, we extended our analysis to include task characteristics as moderators in interaction with age. This moderation analysis tested whether the age‐related improvement in updating performance differed significantly across experimental paradigms. To facilitate interpretation and visualization of task‐specific patterns, we also conducted separate meta‐regressions for each task category. This analytical step addressed the critical question of whether updating development follows a uniform trajectory across different experimental paradigms or whether specific task features reveal distinct patterns of age‐related improvement, thereby providing insights into the cognitive mechanisms underlying developmental change.

Heterogeneity among studies was assessed using Q‐statistics, and the *I* ^2^ statistic quantified the proportion of observed variance reflecting real differences in effect sizes (Higgins et al., 2003). Model comparisons were conducted using the Akaike Information Criterion (AIC) and Bayesian Information Criterion (BIC), both of which balance model complexity against goodness of fit, with lower values indicating better‐fitting models (Akaike, 1974; Bozdogan, 1987; Schwarz, 1978). For evaluation of publication bias, funnel plots were visually inspected to assess the symmetry of effect size distribution against their standard errors (Duval & Tweedie, 2000). The visual inspection was complemented by Egger's regression test, which provides a statistical evaluation of funnel plot asymmetry (Egger et al., 1997).

## RESULTS

### Study selection

The systematic literature search and screening process is illustrated in Figure 1. This rigorous selection process yielded a final sample of 99 studies that satisfied all inclusion criteria. The quality of included studies was evaluated using a custom assessment tool, yielding an average score of 13.45 out of 16 possible points (detailed scores are provided in Supplementary Material Table S2).

**FIGURE 1:**
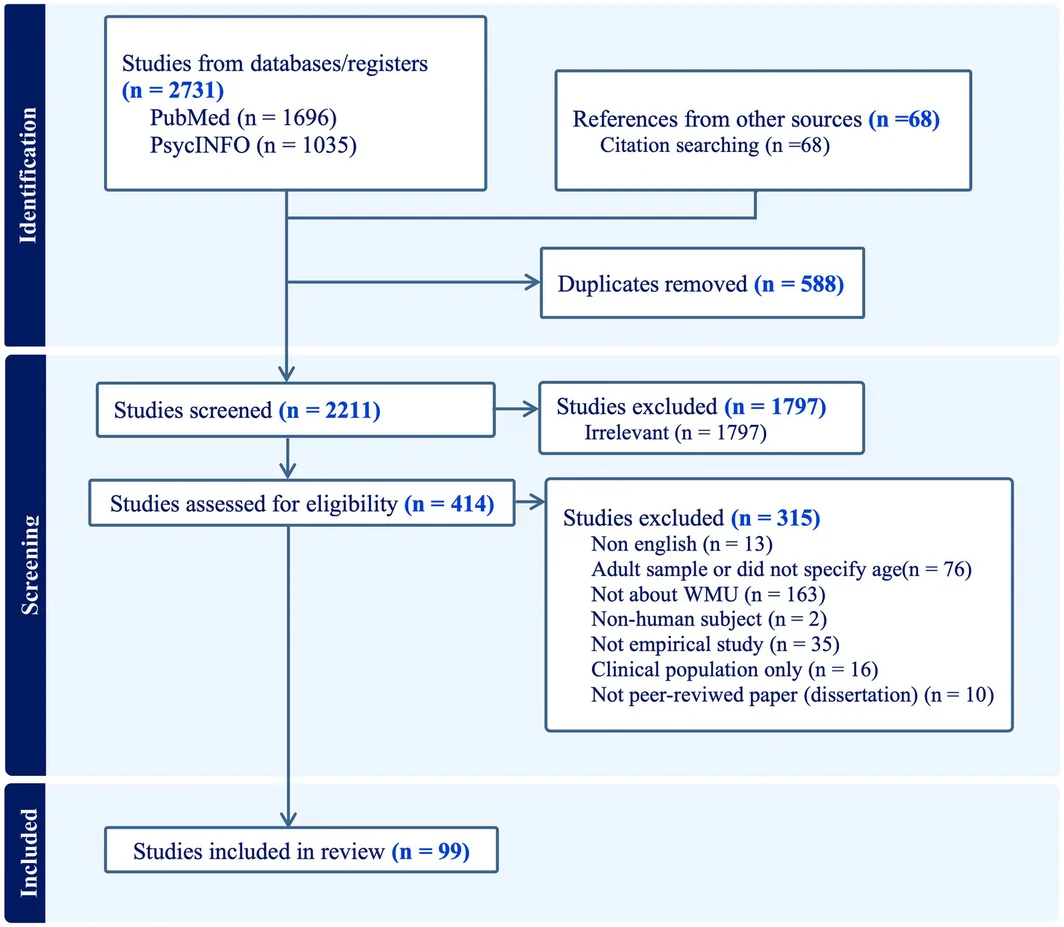
PRISMA Diagram for Study Selection Process. The PRISMA flow diagram illustrates the study selection process. The search yielded 2799 records. After removing 588 duplicates, 2211 records underwent screening. Title/abstract screening excluded 1797 irrelevant studies, leaving 414 for full‐text review. Full‐text screening excluded 315 studies for: language restrictions (*n* = 13), inappropriate samples (*n* = 94), methodological issues (*n* = 163) and publication type (*n* = 45).

These 99 studies yielded 316 datasets comprising 35,858 participants across five age groups: early childhood (3.00–5.99 years; 26 datasets, 1696 participants, *M* = 5.18), early elementary (6.00–8.99 years; 93 datasets, 9704 participants, *M* = 7.43), late elementary (9.00–11.99 years; 112 datasets, 18,148 participants, *M* = 10.33), early adolescence (12.00–14.99 years; 53 datasets, 4587 participants, *M* = 13.04) and late adolescence (15.00–17.99 years; 32 datasets, 1723 participants, *M* = 15.01). The distribution of age and number of studies is shown in Figure 2.

**FIGURE 2:**
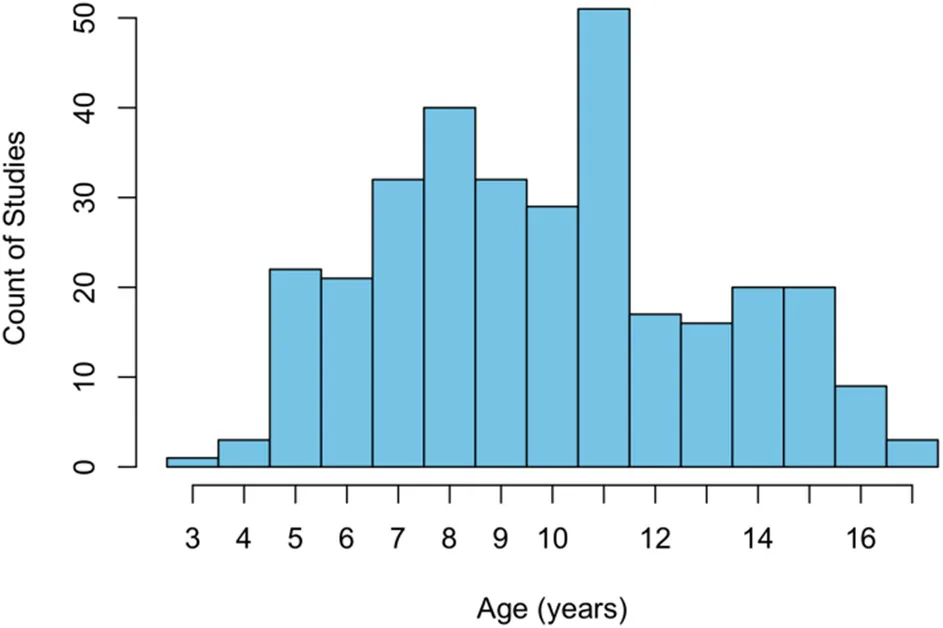
Age Distribution of Study Participants. The bar chart illustrates the age distribution of participants across all included studies. Each bar represents the number of studies that included participants of that specific age. Age 11 shows the highest study count, while age 3 shows the lowest.

### Overall developmental trajectory

The mixed‐effect analysis with age as a moderator revealed a significant positive developmental trend in updating ability (*β* = 0.17, 95% CI [0.14, 0.20], *p* < .0001). This finding indicates a systematic improvement in updating performance across childhood and adolescence, as illustrated in Figure 3. The model accounted for a substantial amount of heterogeneity (*R* ^2^ = 29.87%).

**FIGURE 3:**
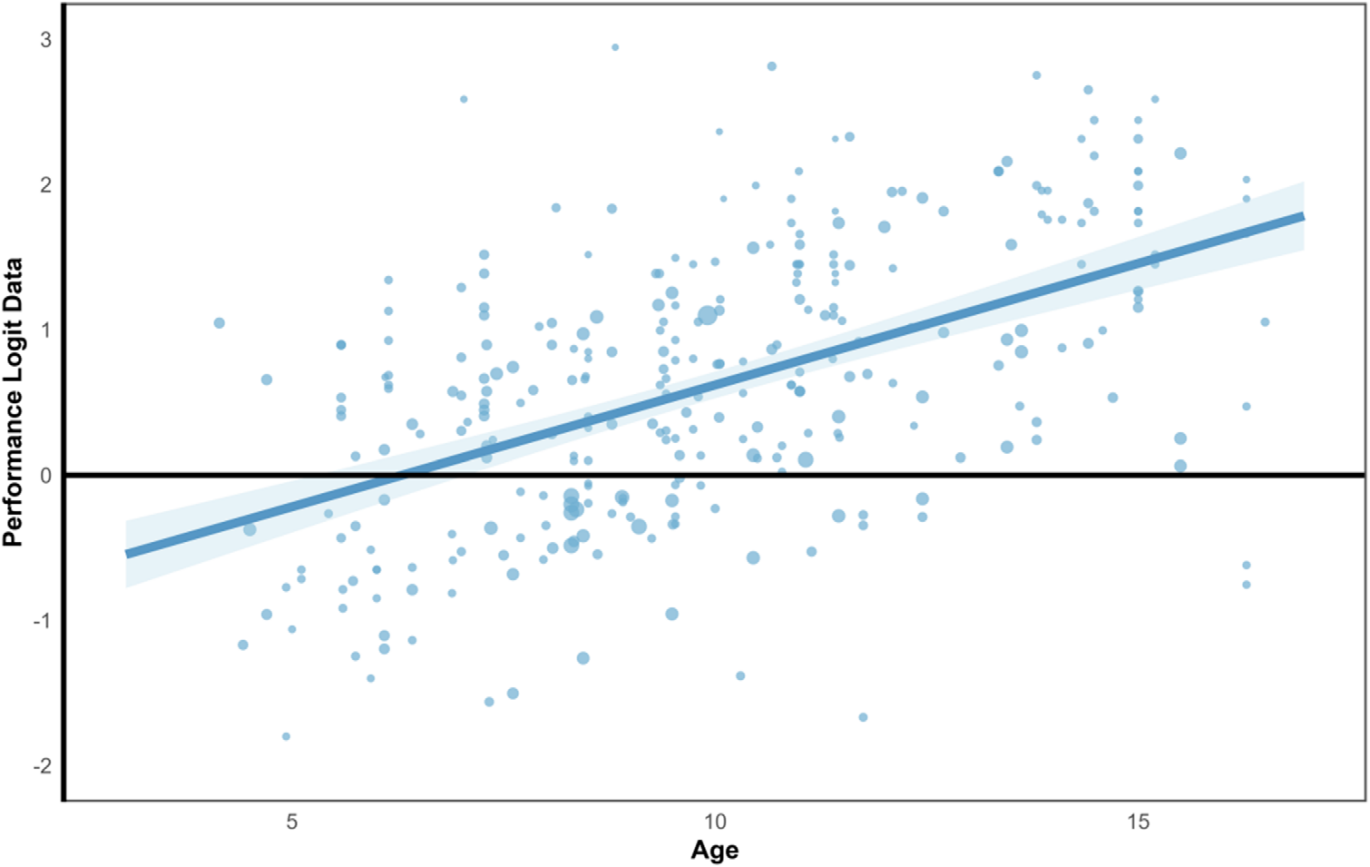
Developmental Trend of Performance with Logit‐Transformed Data. Developmental trend of working memory updating performance with age. Each point represents a study result (logit‐transformed). The solid blue regression line shows significant improvement across childhood and adolescence (*β* = 0.17, *p* < .0001), with 95% confidence interval shown in the shaded area.

Despite the significant age effect, considerable residual heterogeneity remained (τ^2^ = 0.56, SE = 0.054; *I* ^2^ = 95%), suggesting that factors beyond age contribute to variability in updating performance. The test of moderators confirmed age as a significant predictor (QM(1) = 112.83, *p* < .0001). A regression test for funnel plot asymmetry indicated potential publication bias (*z* = 2.81, *p* = 0.005), which we address through sensitivity analyses reported below. Additionally, analysis excluding backward recall tasks continued to show a significant positive developmental trend (*β* = 0.12, *p* < .0001), though with a moderately reduced effect size, confirming the robustness of the developmental trend.

### Age group comparisons

For the age‐group comparison, 6 datasets compared performance between the 3–5 and 6–8 years age group; 51 datasets compared 6–8 and 9–11 years groups; 34 datasets compared 9–11 and 12–14 year groups; and 6 datasets compared 12–14 and 15–17 years groups.

To minimize between‐study variance and examine developmental transitions more precisely, we conducted subgroup meta‐analyses of within‐study age comparisons. These analyses revealed significant improvements across all adjacent age periods, with effect sizes showing a systematic pattern of deceleration with increasing age as shown in Figure 4.

**FIGURE 4:**
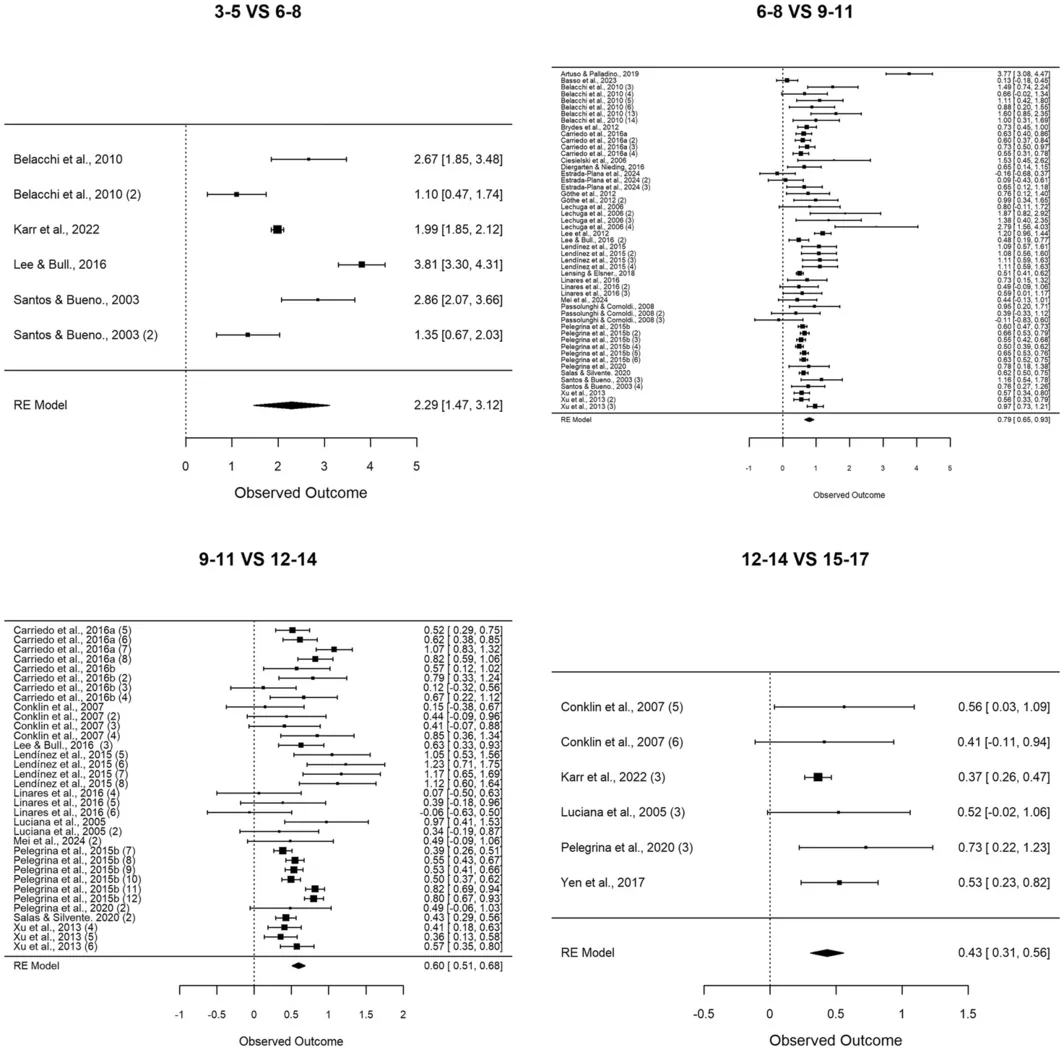
Combined Forest Plots of Age Group Comparisons. Forest plots showing pairwise comparisons between consecutive age groups for working memory updating performance. Each panel displays effect sizes (Cohen's *d*) with 95% confidence intervals for individual studies and random‐effects pooled estimates. Positive values indicate better performance in the older age group. The largest developmental gains occur in early childhood (ages 3–5 vs 6–8: *d* = 2.29), with progressively smaller but significant improvements in later age comparisons (6–8 vs 9–11: *d* = 0.79; 9–11 vs 12–14: *d* = 0.60; 12–14 vs 15–17: *d* = 0.43).

The transition from early childhood to early elementary years (3–5 vs 6–8 years) showed the largest developmental gain (Cohen's *d* = 2.29, 95% CI [1.47, 3.11], *p* < .0001), despite substantial heterogeneity (*I* ^2^ = 93.83%, Q(5) = 66.15, *p* < .0001). This effect size indicates that the average 6–8‐year‐olds perform better than approximately 99% of 3‐5‐year‐olds on updating tasks, highlighting the dramatic cognitive changes during this period.

Subsequent comparisons showed progressively smaller effect sizes: between early and late elementary years (6–8 vs 9–11 years; *d* = 0.79, 95% CI [0.65, 0.93], *p* < .0001), with heterogeneity remaining high (*I* ^2^ = 91.81%, Q(50) = 206.06, *p* < .0001); between late elementary and early adolescence (9–11 vs 12–14 years; *d* = 0.59, 95% CI [0.50, 0.68], *p* < .0001), with a moderate level of heterogeneity (*I* ^2^ = 73%, Q(33) = 108.32, *p* < .0001); and between early and late adolescence (12–14 vs 15–17 years; *d* = 0.43, 95% CI [0.31, 0.55], *p* < .0001), where heterogeneity was low (*I* ^2^ = 12%, Q(5) = 3.33, *p* = 0.64).

These findings suggest a developmental trajectory characterized by substantial early improvements, particularly between preschool and early elementary years, that gradually become more modest in later developmental periods. This pattern of systematically decreasing effect sizes across age comparisons provides additional evidence supporting the negatively accelerated developmental trajectory identified in previous analyses of overall developmental trends.

### Developmental trend modelling

To validate our meta‐regression findings, we conducted additional modelling using the age group comparison data. The meta‐regression revealed a linear relationship in logit space (*β* = 0.1673, SE = 0.016, *z* = 10.62, *p* < .0001, 95% CI [0.14, 0.20]), which transforms to a sigmoid curve in raw performance:

$$\text{Performance}=\frac{1}{1+e^{-\left(-1.0537+0.1673\times \mathrm{age}\right)}}$$

This function describes a rapid early development that gradually slows down. To confirm this pattern, we modelled effect sizes from our age group comparisons. If the meta‐regression accurately captures the developmental trajectory, we would expect effect sizes between age groups to decrease as a function of age, reflecting the diminishing returns implied by the sigmoid curve. We tested three models:

1. Linear
$$d_{i}=\beta_{0}+\beta_{1}\left({\mathrm{Age}}_{\text{diff}}\right)+\varepsilon_{i}$$
2. Quadratic
$$d_{i}=\beta_{0}+\beta_{1}\left({\mathrm{Age}}_{\text{diff}}\right)+\beta_{2}\left({\mathrm{Age}}_{\text{diff}}^{2}\right)+\varepsilon_{i}$$
3. Hyperbolic
$$d_{i}=\frac{1}{\beta_{0}+\beta_{1}\left({\mathrm{Age}}_{\text{diff}}\right)}+\varepsilon_{i}$$

where, *d*_*i*_ represents the effect size for each age group comparison, *Age*_*diff*_ is the age difference between groups and *ε*_*i*_ represents the error term.

The hyperbolic model should theoretically provide the best fit, as it mathematically captures the same developmental pattern as our logit‐transformed meta‐regression.

The model results are shown in Figure 5. All three models showed significant moderation effects, but differed substantially in model fit. The linear model explained *R* ^2^ = 66% of the heterogeneity, the quadratic model explained *R* ^2^ = 87%, and the hyperbolic model provided the best overall fit with an *R* ^2^ of 95%. The hyperbolic model also shows superior information criteria (AIC = −3.18, BIC = −5.02, RMSE = 0.16) compared to quadratic (AIC = 8.32, BIC = 0.31) and linear models (AIC = 8.77, BIC = 4.84). The fitted hyperbolic model was $d=\frac{1}{-0.136+0.212\times \left({\mathrm{Age}}_{\text{diff}}\right)}$, with *β*_*₁*_ = 0.212 (*p* = .018).

**FIGURE 5:**
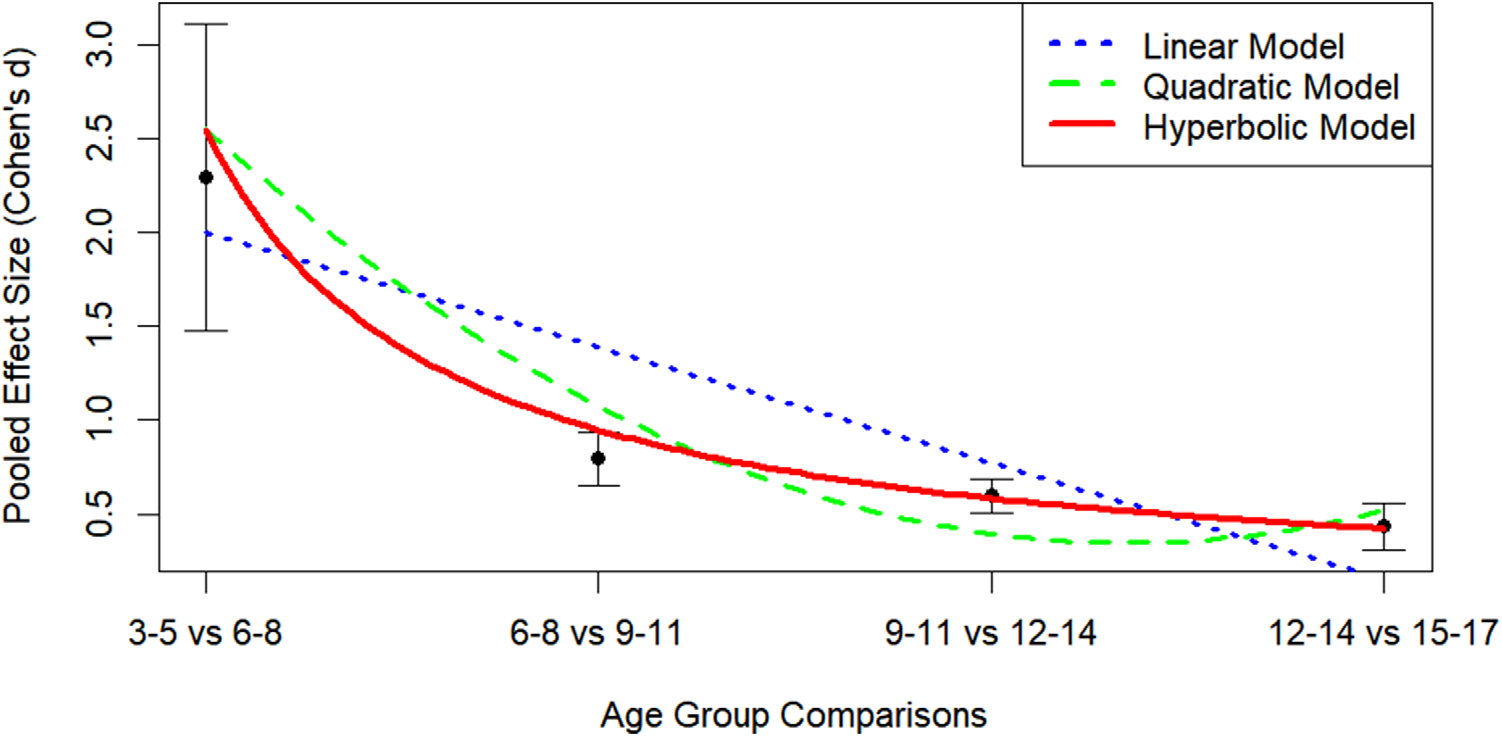
Modelling of Pooled Effect Size across Age Group. Different lines indicate fittings with different models. The dotted blue line shows the best fitting using the linear model. The dashed green line shows the best fitting using the quadratic model. The bold red line shows the best fitting using the hyperbolic model.

The progressive improvement in variance explained (from 66% in the linear model to 87% in the quadratic model, and 95% in the hyperbolic model) strongly supports a non‐linear, decelerating developmental trajectory. The hyperbolic function's superior fit indicates effect sizes decrease following diminishing returns, consistent with our sigmoid trajectory. This convergence across different analytical approaches strengthens our conclusion that WM updating shows rapid early development that gradually plateaus into adolescence, aligning with previous cognitive development research (Ahmed et al., 2022; Brockmole & Logie, 2013; Mous et al., 2017; Tikhomirova et al., 2020).

### Heterogeneity and publication bias

Heterogeneity analyses revealed considerable variation in earlier age comparisons: *I* ^2^ = 94% for ages 3–5 to 6–8 (tau^2^ = 0.95) and *I* ^2^ = 92% for ages 6–8 to 9–11 (tau^2^ = 0.19). Heterogeneity decreases in early adolescence (*I* ^2^ = 73% for ages 9–11 to 12–14; tau^2^ = 0.037) and was low in later adolescence (*I* ^2^ = 12% for ages 12–14 to 15–17; tau^2^ = 0.004). This pattern suggests greater developmental variability or methodological differences in younger children's assessments, with more consistent effects in older participants.

Publication bias evaluation using funnel plot asymmetry tests showed no significant bias for most comparisons: ages 3–5 to 6–8 (*z* = 0.104, *p* = 0.92), ages 9–11 to 12–14 (*z* = 0.087, *p* = 0.93) and ages 12–14 to 15–17 (*z* = 1.53, *p* = 0.13). However, significant asymmetry emerged for ages 6–8 to 9–11 (*z* = 3.67, *p* < .001), indicating potential publication bias in this developmental transition.

The comparisons for ages 3–5 to 6–8 and ages 9–11 to 12–14 both included fewer than 10 studies, which limited the statistical power to detect publication bias (Sterne et al., 2011). The non‐significant results for these groups should therefore be interpreted with caution, as they might reflect insufficient power rather than the absence of bias. Visual inspection of the funnel plot (Figure 6) revealed some asymmetry in the early childhood comparison (ages 3–5 to 6–8), suggesting the possibility of publication bias that our statistical tests were underpowered to detect.

**FIGURE 6:**
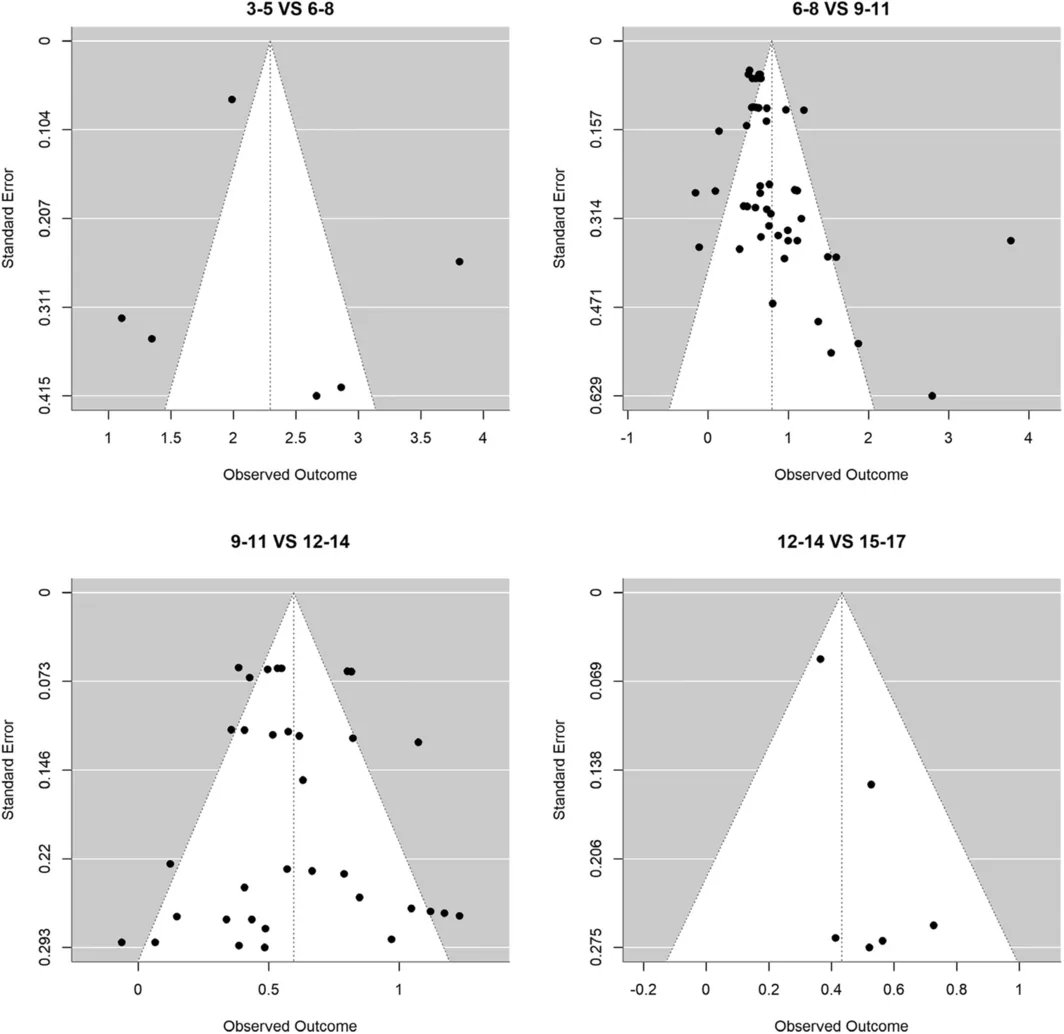
Combined Funnel Plots for Publication Bias Assessment in Age Group Comparisons. Funnel plots assessing publication bias for each age group comparison. Each plot displays effect sizes (x‐axis) against standard errors (y‐axis), with the dashed vertical line indicating the pooled effect estimate. The inverted funnel shape represents the expected distribution under no publication bias, with studies distributed symmetrically around the pooled estimate.

The limited number of studies for the youngest age group (3–5 years), combined with greater variability in individual study estimates, indicated greater uncertainty in effect size estimates for this developmental period. This uncertainty likely reflected both the smaller number of available studies and the inherently greater individual differences in WM updating performance during early childhood (Garon et al., 2008). Therefore, the exact magnitude of developmental gains observed during early childhood should be interpreted with particular caution. Regardless, the observed developmental pattern is consistent with theoretical expectations and prior research (Karr et al., 2022), and the high heterogeneity (*I* ^2^ = 94%) suggested genuine variability rather than systematic bias.

### Task‐specific developmental patterns

Analysis of 316 datasets across five task categories revealed distinct developmental trajectories. We chose to use backward recall tasks as the reference category, the analysis showed steep developmental improvements (*β* = 0.21, 95% CI [0.15, 0.27], *p* < .001). This choice was based on two reasons: (1) backward recall task represents the most commonly studied paradigm in our dataset (84 datasets), providing stable baseline estimates; and (2) it exhibits the steepest developmental trajectory, making it a sensitive benchmark for detecting relative differences in other tasks' developmental patterns. Keep track tasks demonstrated comparable developmental trajectories (Age × Task interaction: *β* = −0.02, *p* = .599), with similarly strong age‐related gains (*β* = 0.19, 95% CI [0.11, 0.27], *p* < .001). In contrast, n‐back tasks showed significantly flatter developmental slopes than backward recall tasks (Age × Task interaction: *β* = −0.11, *p* = .010), with more modest age associations (*β* = 0.10, 95% CI [0.01, 0.18], *p* = .025). Running memory tasks demonstrated intermediate improvements (*β* = 0.14, 95% CI [0.05, 0.23], *p* = .003) which did not differ significantly from backward recall tasks (Age × Task interaction: *β* = −0.07, *p* = .191). Most notably, selective updating tasks exhibited significantly flatter trajectories than backward recall (Age × Task interaction: *β* = −0.24, *p* < .001), with minimal developmental gains (*β* = −0.03, 95% CI [−0.09, 0.04], *p* = .400). These findings indicate that the development of updating abilities varies substantially by paradigms, as depicted in Figure 7. Specifically, backward recall tasks and keep track tasks show rapid age‐related improvements, whereas n‐back and selective updating tasks exhibit flatter developmental trajectories. This variability underscores the importance of considering task‐specific characteristics when evaluating updating development and suggests that individual differences in strategy use or task familiarity may further shape these developmental patterns.

**FIGURE 7:**
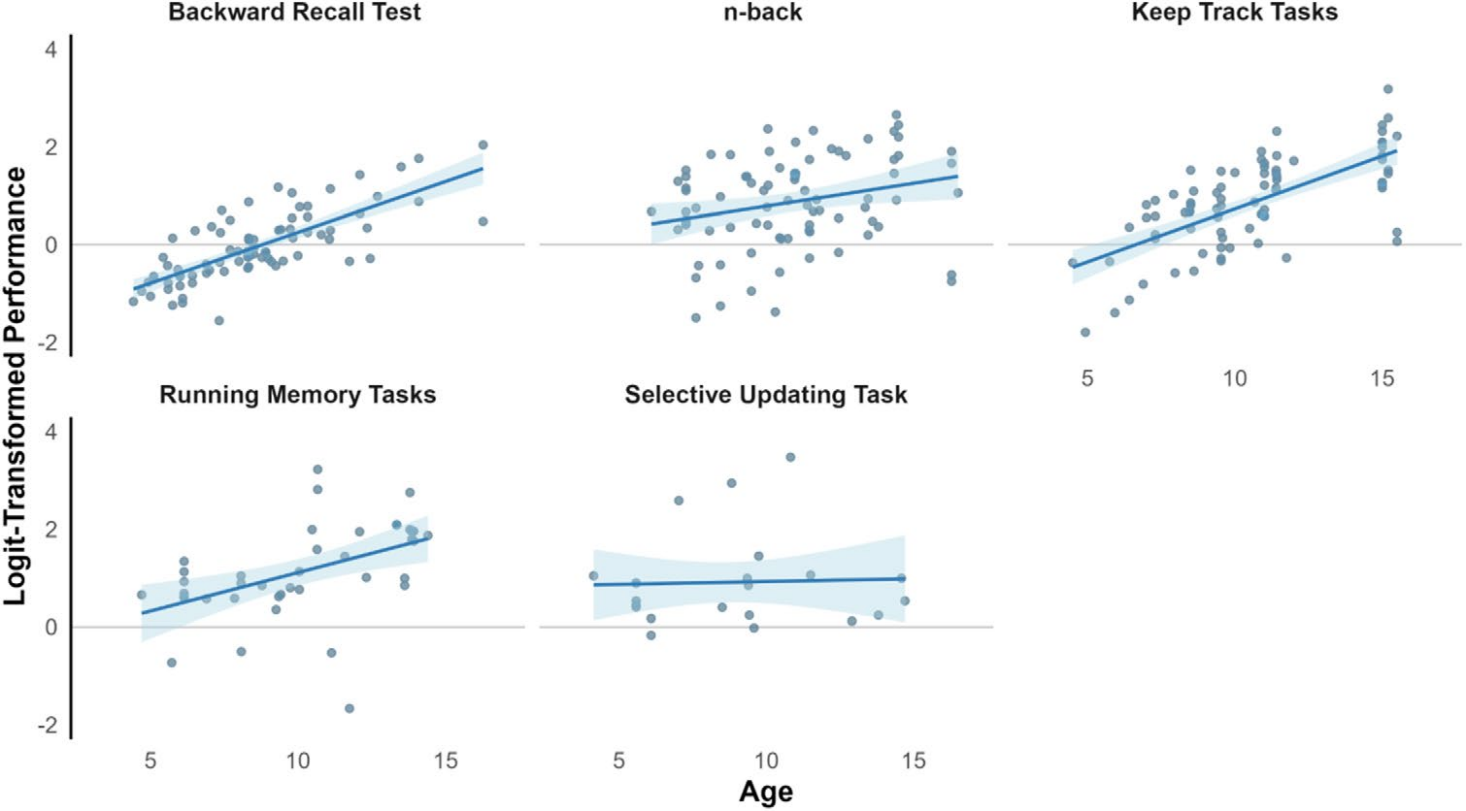
Developmental Trajectories of Working Memory Updating by Task Type. Developmental trajectories of working memory updating performance across five task categories. Each panel shows logit‐transformed performance plotted against age, with individual effect sizes represented as points and fitted regression lines with 95% confidence intervals. Clear developmental improvements are evident for Backward Recall, n‐back, Keep Track and Running Memory tasks, while Selective Updating tasks show minimal age‐related change. The steepest developmental gains occur in Keep Track and Running Memory tasks, suggesting these may be particularly sensitive to developmental changes in updating abilities.

### Sensitivity analysis

A sensitivity analysis was conducted to examine whether the study quality affected the developmental trend. Results showed that the study quality did not significantly moderate the age effect (*p* = .278), and the developmental trend remained unchanged when controlling for quality (*β* = 0.161 vs *β* = 0.164 without control), confirming the robustness of our findings.

The comparison between ages 6–8 and 9–11 years warranted particular attention due to the high heterogeneity (*I* ^2^ = 92%) and significant funnel plot asymmetry (*z* = 3.67, *p* < .001), suggesting potential publication bias in this age range. This pronounced heterogeneity likely reflects the substantial individual differences that emerge during primary school years, as children experience varying rates of cognitive maturation and educational exposure. The primary school period represents a critical transition from intuitive to more formal learning strategies, with considerable variability in how children integrate executive functions into academic tasks (Hammerstein et al., 2019).

To investigate potential sources of bias and heterogeneity, sensitivity analyses were conducted. Diagnostic metrics identified two studies as potential outliers with high studentized residuals (rstudent = 8.14 and 2.68), moderate influence on fitted values (dffits = 1.87 and 0.31) and Cook's distance of 1.81 and 0.09, respectively. Excluding these outlying studies resulted in a modest reduction in the pooled effect size from 0.79 to 0.68, and a substantial reduction in heterogeneity (*I* ^2^ from 92% to 68%). Funnel plot asymmetry was also reduced (*z* = 2.88, *p* = 0.004), although some asymmetry remained, indicating that additional factors may contribute to the observed pattern.

To explore the sources of heterogeneity more systematically, meta‐regression analyses were conducted with task category moderators. The addition of task type as moderators significantly improved model fit (QM = 11.90, *p* = 0.018) and substantially reduced heterogeneity (*I* ^2^ = 47%). The task moderators accounted for *R* ^2^ = 55% of the total heterogeneity, representing a moderate‐to‐large reduction in unexplained variance. However, significant residual heterogeneity remained even after accounting for task type (QE = 89.17, *p* < 0.001), suggesting that additional unmeasured factors contribute to between‐study differences.

Importantly, the overall conclusions of the analysis remained consistent across all sensitivity analyses, suggesting that the results are robust to the influence of individual studies. The remaining heterogeneity likely reflects genuine developmental diversity rather than methodological artefacts underscoring the substantial individual differences in updating ability development during the primary school years. This pattern has important educational implications, suggesting that instructional approaches may need differentiation to accommodate the wide range of updating abilities present in typical classrooms (Hu et al., 2023).

Interestingly, heterogeneity decreased substantially in later developmental periods, with moderate heterogeneity during early adolescence (*I* ^2^ = 73% for ages 9–11 to 12–14) and low heterogeneity in later adolescence (*I* ^2^ = 12% for ages 12–14 to 15–17). This pattern suggests that age‐related effects become more consistent as children approach late adolescence, indicating greater developmental stability in updating abilities during this period.

To examine whether our developmental findings were robust, we conducted additional analyses excluding backward recall tasks. The meta‐regression excluding backward recall (*k* = 232) showed a somewhat attenuated but still highly significant developmental trend (*β* = 0.12, SE = 0.019, *p* < .0001), compared to *β* = 0.17 in the main analysis. This represents a 29% reduction in slope, and the model accounted for substantially less heterogeneity (*R* ^2^ = 17.38% vs 30.19% in the main analysis). Despite this reduction, substantial residual heterogeneity persisted (*I* ^2^ = 94.64%, τ^2^ = 0.60), and evidence of publication bias remained significant (*z* = 3.67, *p* = 0.0002). Furthermore, the Age group comparisons excluding backward recall tasks showed a similar pattern of decreasing effect sizes with age (3–5 vs 6–8: *d* = 2.81; 6–8 vs 9–11: *d* = 0.84; 9–11 vs 12–14: *d* = 0.60; 12–14 vs 15–17: *d* = 0.43). The hyperbolic model remained the best‐fitting model when backward recall tasks were excluded, further supporting the robustness of the developmental trajectory (see detailed statistical results in Supplemental Material Figures S5‐S8).

## DISCUSSION

The meta‐analysis examined the developmental trajectory of WM updating from early childhood through late adolescence, analysing 316 datasets from 99 studies comprising 35,858 participants from 3 to 17 years old. The results revealed a systematic improvement in updating performance across development, with the rate of improvement gradually decreasing with age. The mixed‐model meta‐regression indicated a significant positive developmental trend, demonstrating substantial developmental gains in updating ability across childhood and adolescence.

### Developmental trajectory

Our analyses revealed a negatively accelerated developmental pattern characterized by substantial improvement in early childhood that levels off into late adolescence. This trajectory emerged consistently across two independent analytical approaches: the mixed‐effects meta‐regression modelling individual trial data in logit space (*β* = 0.1673 logit units per year) and the hyperbolic function fitted to age‐group effect sizes (*d* = 0.212/Age‐0.136).

The hyperbolic model explained 95% of the variance in age‐group effect sizes, substantially outperforming both linear (*R* ^2^ = 66%) and quadratic (*R* ^2^ = 87%) models. When transformed from logit space, both approaches yield a sigmoid curve, showing rapid early development that gradually plateaus, consistent with established theories of cognitive development and neural maturation patterns (Crone et al., 2006; Luna et al., 2015).

The most substantial developmental gains occur between the youngest age groups. The transition from 3–5 to 6–8 years shows the largest increase in predicted performance (Δ = 0.12) and the most pronounced effect size (*d* = 2.29), indicating that the average 6–8‐year‐old performs better than approximately 99% of 3–5‐year‐olds. This dramatic improvement reflects a critical period of neural maturation in the prefrontal regions essential for updating ability (Rose et al., 2011). However, this substantial effect should be interpreted with caution due to the limited number of available datasets (*n* = 6) and considerable heterogeneity among studies (*I* ^2^ = 93.83%).

Subsequent age transitions show progressively smaller gains, confirming the negatively accelerated developmental trajectory. This pattern is consistent with a body of longitudinal and cross‐sectional research on WM and executive function (Ahmed et al., 2022; Baum et al., 2017; Friedman & Robbins, 2022; Huizinga et al., 2006; Poon, 2018; Van Leijenhorst et al., 2007; Zelazo & Carlson, 2012), where core components such as inhibitory control, cognitive flexibility and WM updating continue maturing well into adolescence (Poon, 2018; Zelazo & Carlson, 2012). This developmental trajectory is accompanied by significant advances in the control and flexible use of WM information, as EF components including inhibitory control, cognitive flexibility, proactive control and planning co‐develop and become increasingly integrated during this period (Friedman & Robbins, 2022; Huizinga et al., 2006; Zelazo & Carlson, 2012). These behavioural changes are underpinned by neural maturation, particularly within the prefrontal cortex and its associated networks (Friedman & Robbins, 2022). For example, increased segregation and efficiency of structural brain networks in the prefrontal cortex have been closely linked to improvements in executive performance across development (Baum et al., 2017).

#### Task‐specific developmental patterns

The observed variations in task‐specific developmental trajectories can be understood through the lens of their varying dependence on WM capacity versus executive control processes. Updating performance depends fundamentally on the capacity to maintain relevant information in an active state, though the updating process itself involves executive control for selecting, enhancing and discarding information (Ecker et al., 2010; Panesi et al., 2022). The narrow capacity limits of young children constrain their performance particularly on tasks requiring simultaneous maintenance of multiple items or categories, whereas older children and adolescents possess sufficient capacity to deploy more sophisticated updating strategies (Cowan, 2001; Morra et al., 2018). This interaction could explain our finding that tasks that place higher demands on capacity show steeper developmental trajectory, while those with higher emphasis on executive control with lower capacity demands show more gradual or varying patterns, with task‐ specific features further modulating these trajectories.

Specifically, our analysis identified that the backward recall tasks exhibited the most pronounced age‐related improvement, indicating robust developmental gains. Similar positive trends were observed in the keep track task, showing a steady increase in performance with age. The n‐back task and running memory task, though less steep, both demonstrated a moderate upward trajectory. In contrast, the selective updating task did not show significant age‐related improvement; performance remained relatively stable.

The distinctive pattern for backward recall tasks may be attributed to several developmental factors. First, the backward recall task imposes the highest WM capacity load. Rather than presenting a uniform memory load to all participants, these tasks employ an escalating difficulty design that systematically identifies each individual's capacity ceiling (Gathercole & Pickering, 2000). This threshold‐seeking approach ensures that performance is measured at the boundaries of ability rather than within comfortable operating ranges, thereby maximizing sensitivity to age‐related capacity expansion. Combined with the cognitive demand of simultaneously storing and manipulating information, backward recall presents a particularly steep challenge for younger children and effectively captures developmental trajectories in WM capacity (Alloway & Alloway, 2013; Hilbert et al., 2015; Wang & Gathercole, 2013). These tasks primarily assess the ability to recall information in reverse order, which requires mental transformation of sequential representations‐a process that becomes more efficient with age as children develop more sophisticated strategies for serial order processing (Li & Lewandowsky, 1995; St Clair‐Thompson, 2010). The steeper developmental trajectory for backward recall tasks compared to pure updating tasks suggests they are particularly sensitive to the expansion of working memory capacity across childhood.

Second, backward recall tasks require an inhibition component: individuals must suppress the prepotent response to recall items in their original order and instead reconstruct them in reverse (Engle, 2002; Miyake et al., 2000). This mental manipulation likely depends on the maturation of the dorsolateral prefrontal cortex, which continues to develop well into adulthood (Crone et al., 2006). The ability to hold items in mind while simultaneously reordering them represents a form of dual‐task coordination that improves gradually with age as executive control mechanisms become more efficient (Crone et al., 2006; Hilbert et al., 2015; Moriguchi & Hiraki, 2013). Additionally, the ability to mentally manipulate information draws on temporal position monitoring and interference control, which shows protracted development throughout childhood and adolescence (Buttelmann & Karbach, 2017; Davidson et al., 2006).

Third, the cognitive strategies underlying backward recall performance differ potentially across developmental stages. Young children's performance might be constrained by capacity limitation such that they have difficulty maintaining even two or three items while simultaneously manipulating their order. As capacity expands through middle childhood, children become able to maintain longer sequences, but may still employ relatively simple strategies such as mentally scanning a maintained list in reverse order (Li & Lewandowsky, 1995). Only in later childhood and adolescence do some individuals begin to adopt more sophisticated recursive updating strategies, such as immediately repeating the final item, then mentally rehearsing the entire list to retrieve the penultimate item and repeating this process recursively.

Our sensitivity analyses provide some insight into this question. When backward recall tasks were excluded, the developmental trend remained significant (*β* = 0.12) but was attenuated by 29%, and the model explained substantially less heterogeneity (*R* ^2^ = 17.38% vs 30.19%). This suggests that backward recall tasks capture developmental variance beyond what is measured by the four more conventional updating tasks, but whether this additional variance reflects capacity expansion, updating processes or both remains ambiguous.

In contrast to backward recall tasks, the other conventional updating tasks showed greater variability in developmental patterns. This suggests that while all updating tasks depend on capacity to some extent (Ecker et al., 2010; Panesi et al., 2022), their developmental trajectories are more strongly influenced by task‐specific control demands and strategic factors. This heterogeneity likely reflects their diverse cognitive demands. Specifically, n‐back tasks involve recognition memory and sustained attention; keep track tasks require simultaneous category monitoring; running memory tasks depend on recency effects and retrieval strategies; and selective updating tasks require controlled inhibition of specific memory positions. We discuss each of the tasks in more detail below.

Keep track tasks showed pronounced age‐related improvements (*β* = 0.19) with a slightly smaller but statistically comparable slope to backward recall tasks (*β* = 0.19 vs 0.21, *p* = .599), reflecting their unique position as bridging capacity‐dependent and executive control‐dependent updating. These tasks require updating specific categorical information while discarding irrelevant details, drawing on categorization abilities that develop significantly during childhood (Friedman et al., 2008). Indeed, research has shown that categorization ability becomes more sophisticated at around age 7, with both categorization strategies and mechanisms evolving over time (Deng & Sloutsky, 2015; Deng & Sloutsky, 2016). Notably, keep track tasks place particularly high demands on working memory capacity, especially when three or more categories must be monitored simultaneously. Younger children's limited capacity severely constrains their ability to maintain even two categories simultaneously, whereas older children and adolescents have sufficient capacity to deploy executive control strategies effectively. The robust development in keep track task performance likely reflects improvements in multiple components including retrieval strategies (Imbo & Vandierendonck, 2007), selective attention (Hanania & Smith, 2010) and WM capacity itself.

The n‐back task, another widely used task, displayed weaker and more variable associations with age, possibly reflecting its unique demands on recognition memory and continuous monitoring processes that differ from other updating tasks. The binary response format of most n‐back tasks creates a relatively high chance level (50%) (Herff et al., 2013), which poses challenges for observing developmental gains and makes near‐chance performance difficult to interpret without correction for guessing rates. The modest improvement across age groups can be explained by the dual‐process nature of n‐back tasks, which involve both familiarity‐based recognition that develops early and recollection‐based processes that become more pronounced during later childhood (Ghetti & Bunge, 2012; Jaeggi et al., 2010; Kane et al., 2007). Additionally, n‐back tasks impose substantial demands on interference control, as participants must distinguish between currently relevant items and previously relevant but now irrelevant items (Kane et al., 2007). The development of interference control mechanisms may follow a different trajectory compared to other executive function components (Bunge & Wright, 2007). Furthermore, the continuous nature of n‐back tasks requires sustained attention, which may be influenced by motivational factors that vary across development (Unsworth & Robison, 2020). Nonetheless, the variable patterns observed in n‐back tasks may partly reflect differences in capacity demands: while the 1‐back version places minimal load, the 2‐back and higher levels impose substantial demands on both WM capacity and updating execution. This load‐dependent variability, together with the task's unique cognitive profile, may explain why n‐back shows less consistent developmental patterns than other updating measures.

The other two tasks, running memory tasks and selective updating tasks, demonstrated positive but more gradual developmental patterns. Running memory tasks demonstrated positive but more gradual developmental patterns, reflecting their moderate dependence on capacity alongside strategic and perceptual factors. These tasks involve continuous updating of a memory set as new items are presented, depending on both storage capacity and efficient removal of no‐longer‐relevant information, which processes that appear to develop gradually across childhood and adolescence (Hasher et al., 2008; Williams, 1999). As Cowan (2001) noted, running memory depends on capacity to maintain the required number of recent items, but this dependence is mitigated by recency effects and retrieval strategies that can compensate for limited capacity. Additionally, running memory tasks require ‘updating efficiency’, which involves both processing speed and precision in recalling information. This efficiency improves with age as processing speed increases and attentional control becomes more refined (Huizinga et al., 2006). The development of proactive interference resistance, which allows children to overcome the intrusion of previously relevant information, may also contribute to the observed age‐related improvements (Loosli et al., 2016). The interplay between the capacity, speed and interference control development may produce more variable developmental trajectories compared to tasks with more unified cognitive demands.

Finally, the selective updating task demonstrated the flattest developmental trajectory, suggesting minimal age‐related gains. However, firm conclusions about this pattern are substantially constrained by methodological limitations. Only 11 studies were included in this category, limiting statistical power to detect developmental effects. More critically, existing studies employ different task paradigms tailored to their target age groups, making direct developmental comparisons challenging. The lack of standardized versions across ages may create artificial floor or ceiling effects at different developmental periods. Given these limitations, we propose two tentative interpretations of the flat trajectory, both requiring further empirical investigation. First, the pattern may reflect genuine developmental characteristics of selective updating as a construct. Selective updating task emphasizes executive control over capacity demands: it requires controlled inhibition of specific memory positions and enhancement of others, but typically involves maintaining only a small set of items (often 3–4), which is within even young children's capacity limits. Unlike keep track tasks that require simultaneous monitoring of 2–4 categories, selective updating's lower capacity demands may allow even young children to engage the task meaningfully. The flat pattern may reflect that the executive control processes required, attentional filtering and top‐down modulation, develop relatively early and plateau by middle childhood (Rueda et al., 2004), or alternatively that these processes show high individual variability that obscures age trends (Friedman & Robbins, 2022). The observed variability may reflect individual differences in the development of prefrontal regions involved in selective attention and cognitive control, as well as in metacognitive abilities that support monitoring and regulation of updating processes (Roebers, 2017). Alternatively, the flat trajectory may be a methodological artefact rather than a genuine developmental pattern. If tasks are inappropriately difficult for younger children (producing floor effects) or too easy for older participants (producing ceiling effects), age‐related improvements would be masked. The heterogeneity in task implementations across the 11 studies prevents systematic evaluation of whether such effects are present.

As depicted in Figure 7, these results indicate that the development of updating ability during childhood and adolescence varied substantially by tasks. The variability observed across tasks, particularly in running memory and n‐back tasks, underscores the potential influence of individual differences on developmental trajectories. Recent research demonstrated that children employed a range of memory strategies when performing serial recall and updating tasks, with both the adaptivity and effectiveness of these strategies increasing with age (Poloczek & Jarrold, 2025). Furthermore, individual differences in baseline WM capacity can shape how effectively children utilize these strategies, thereby influencing overall task performance (Congdon, 2024). Prior exposure to similar cognitive tasks, whether through formal education or targeted cognitive training, also plays a significant role: familiarity with task demands can reduce cognitive load and facilitate more efficient processing (Holmes et al., 2009; Jaeggi et al., 2010). Thus, these individual differences in both strategy adoption and task experience contribute to the wide range of developmental patterns observed, highlighting the importance of considering such factors when interpreting age‐related changes in updating ability. However, smaller datasets for running memory tasks (42 datasets) and selective updating tasks (25 datasets) may contribute to wider confidence intervals compared to other tasks.

In sum, our findings support a framework in which updating task performance depends on both working memory capacity and executive control processes, with their relative contributions varying across tasks. Tasks that utilize higher capacity demands (backward recall, keep track with multiple categories) show steeper developmental trajectories, reflecting the substantial expansion of WM capacity from childhood through adolescence. Tasks with lower capacity demands but higher executive control requirements (selective updating) show flatter or more variable patterns, potentially reflecting earlier development of basic control processes or high individual variability. Tasks involving intermediate demands or multiple sources of impurity (n‐back at higher loads, running memory) show moderate and variable trajectories. This framework suggests that developmental researchers should carefully consider the type and the amount of cognitive demands employed in the tasks when selecting updating measures and interpreting age‐related changes. Future research would benefit from systematic manipulation of capacity demands within updating paradigms to better disentangle these developmental processes. Specifically, studies could employ parametric designs that vary set size (e.g. 2‐item vs 4‐item keep track tasks) or updating frequency (e.g. number of swaps in selective updating tasks) within the same participants across different age groups. Such designs would allow researchers to model developmental trajectories separately for capacity‐limited versus executive control‐limited performance, potentially revealing whether younger children's updating difficulties primarily reflect insufficient storage capacity or immature control processes. Additionally, combining behavioural paradigms with neuroimaging or computational modelling approaches could illuminate whether age‐related improvements reflect changes in capacity parameters, control efficiency or both.

#### Theoretical and practical implications

Our meta‐analysis revealed typical developmental trajectories of WM updating from early childhood to adolescence. This can help educators and caregivers set realistic expectations and provide appropriate support for children who are still maturing in these abilities. Specifically, the transition periods identified in our analysis align with typical educational transitions (kindergarten entry, transition to middle school), suggesting the need for appropriate scaffolding during these periods when updating demands increase. Since WM updating develops alongside concurrent changes in other cognitive skills and brain structures, younger children may benefit from instructional approaches that reduce updating demands while their cognitive systems continue to mature.

Furthermore, the divergent developmental patterns observed across different updating tasks highlight the multifaceted nature of updating ability. Specifically, our findings show distinct task‐specific developmental trajectories: tasks that primarily depend on capacity limitations (e.g. backward recall tasks) exhibit steeper age‐related improvements, whereas those requiring greater engagement on executive control processes (e.g. running span tasks) display more minimal or variable developmental change. This variability underscores the importance of considering task‐specific demands when assessing the development of WM updating across age groups and selecting age‐appropriate paradigms. For instance, because different components of updating may follow distinct maturation timelines, researchers studying younger children might consider using tasks that rely more heavily on capacity‐based updating to obtain a more sensitive measure of developmental changes. In contrast, for older children and adolescents, employing paradigms that engage more executive control‐related components (e.g. monitoring, planning, switching) may help reveal a more accurate estimation of their updating ability.

The findings directly address questions about the construct validity of including backward recall in updating assessments. Our sensitivity analyses demonstrate three key points: First, backward recall tasks are valid measures of age‐sensitive working memory manipulation, showing robust and consistent developmental patterns. Second, they tap into somewhat different processes than the other updating tasks, with stronger dependence on capacity expansion. Third, the persistence of significant developmental effects even after excluding backward recall confirms that updating processes continue to develop beyond simple capacity expansion, though capacity clearly plays a foundational role.

#### Recommendations and future directions

Our meta‐analysis findings direct several recommendations for future WM updating research in developmental populations. Firstly, the scarcity of research in the youngest age range stems from methodological challenges inherent in studying young children, including difficulties with task compliance, short attention spans and the need for developmentally appropriate paradigms that can reliably assess updating processes (Panesi & Morra, 2019). Tasks that measure updating are especially demanding because they require children to attend to, remember, and constantly manipulate the monitored information as new information comes in. Young children's short attention spans can make it difficult for them to fully engage with these tasks (Moyer et al. 1954), which in turn affects the validity of the measurements. If children struggle to stay on task or do not comply with instructions, their performance may not accurately reflect their actual cognitive levels. As a result, developing tasks that are both engaging and appropriate for young children is essential for obtaining reliable measures of their cognitive skills. Additionally, longitudinal designs tracking individual trajectories across this critical transition are particularly resource‐intensive, requiring substantial temporal and financial demands (Caruana et al., 2015). Future research should prioritize developing age‐appropriate updating tasks specifically designed for young children, employing longitudinal designs spanning this critical developmental window and potentially integrate neuroimaging techniques to better characterize the neural mechanisms underlying this accelerated developmental period.

Relatedly, when choosing age‐appropriate tasks, researchers should consider: (1) difficulty calibration to avoid ceiling/floor effects, (2) cognitive demands matching developmental capabilities and attention spans and (3) developmental sensitivity to detect meaningful changes within target age ranges. Critically, researchers must rigorously evaluate whether tasks actually measure updating ability as theoretically defined. Our screening revealed several studies mislabelling tasks as ‘updating’ measures despite lacking key components (e.g. requiring only storage without active manipulation), contributing to inconsistent findings and conceptual confusion in the literature. Clear operational definitions and careful task validation are essential for advancing theoretical understanding of updating development.

Future research should also investigate how educational environments can be optimized to accommodate children's developing updating abilities. Rather than solely focusing on interventions to improve children's skills, research should examine how curriculum design and instructional methods can be adapted to match developmental capabilities, particularly during the transition periods identified in our analysis. This includes investigating what supports and scaffolding best accommodate individual variability in updating development while maintaining appropriate developmental expectations.

The substantial heterogeneity observed in our analyses, particularly during the primary school years, highlights the need for research examining individual differences in updating development. Several critical questions remain regarding the factors influencing the wide variability in updating performance among children of the same age and whether the difference in observation reflects different developmental trajectories and individual differences or measurement error and contextual factors. Longitudinal designs tracking children across multiple time points would illuminate whether updating abilities develop continuously or show periods of rapid change, and whether early individual differences predict later cognitive and academic outcomes. Such research should investigate potential moderators including socio‐economic status, language exposure, educational quality and neurodevelopmental factors that may shape updating trajectories.

Given WM updating's critical role in academic achievement, surprisingly few studies have directly examined how updating development supports educational outcomes across childhood. Future research should investigate specific mechanisms through which updating abilities facilitate learning in different academic domains. For instance, does updating support mathematics learning primarily through problem‐solving flexibility, or through retention of intermediate calculation steps? In reading comprehension, does updating enable integration of new information with prior text, or monitoring of comprehension accuracy? Understanding these domain‐specific relationships would inform both theoretical models and educational interventions.

Another critical direction for future research involves addressing the growing prevalence of learning difficulties and neurodevelopmental disorders, which often manifest through WM storage and updating deficits (Bombonato et al., 2024; Holmes et al., 2014). Educational disparities have been linked to differences in executive functions, as problem‐solving and reasoning require dynamic updating of information in response to changing task demands (Alloway et al., 2009; Peng et al., 2013). Developing sensitive, developmentally appropriate assessments and interventions for WM updating deficits will be essential for supporting at‐risk children and reducing educational inequalities.

## AUTHOR CONTRIBUTIONS

**Ye Song:** Conceptualization; methodology; investigation; data curation; formal analysis; writing – original draft; writing – review and editing; visualization; validation. **Chen Cheng:** Conceptualization; investigation; validation; supervision; writing – original draft; writing – review and editing.

## Supporting information


**Table S1.** Quality index criteria and scoring. Quality assessment criteria with scoring range 0–2 for each dimension. Maximum possible score = 16. NA = not applicable.
**Table S2.** Scores for individual studies included in the analysis. Individual quality scores for each study based on criteria in Table 1 (0 = not reported/not standardized, 1 = partially reported/standardized, 2 = fully reported/standardized). Total score = sum of all criteria (maximum = 16).
**Figure S1.** Forest plot for comparison between age groups 3–5 and 6–8 years. Forest plot showing effect sizes (Cohen's *d*) and 95% confidence intervals for individual studies comparing working memory performance between younger (3–5 years) and older (6–8 years) children. The diamond represents the pooled random‐effects estimate (*d* = 2.29, 95% CI [1.47, 3.12]). Positive values indicate better performance in the older age group.
**Figure S2.** Forest plot for comparison between age groups 6–8 and 9–11 years. Forest plot comparing working memory performance between age groups 6–8 and 9–11 years. Pooled random‐effects estimate: *d* = 0.79, 95% CI [0.65, 0.93].
**Figure S3.** Forest plot for comparison between age groups 9–11 and 12–14 years. Forest plot comparing working memory performance between age groups 9–11 and 12–14 years. Pooled random‐effects estimate: *d* = 0.60, 95% CI [0.51, 0.68].
**Figure S4.** Forest plot for comparison between age groups 12–14 and 15–17 years. Forest plot comparing working memory performance between age groups 12–14 and 15–17 years. Pooled random‐effects estimate: *d* = 0.43, 95% CI [0.31, 0.56].
**Figure S5.** Developmental trajectory excluding backward recall tasks. This sensitivity analysis excludes all backward recall tasks. The meta‐regression model showed significant age‐related improvement (*β* = 0.122, SE = 0.019, *p* < .001), with age accounting for 17.38% of variance in performance (*R* ^2^ = 17.38%). Substantial heterogeneity remained after accounting for age (*I* ^2^ = 94.64%, τ^2^ = 0.60).
**Figure S6.** Combined forest plots excluding backward recall tasks. Top left panel: Ages 3–5 versus 6–8 (*k* = 3); Top right panel: Ages 6–8 versus 9–11 (*k* = 38); Bottom left panel: Ages 9–11 versus 12–14 (*k* = 31); Bottom right panel: Ages 12–14 versus 15–17 (*k* = 3). Random‐effects meta‐analyses across four age comparisons, excluding backward recall tasks (total *k* = 75 effect sizes). Effect sizes show systematic developmental deceleration: Ages 3–5 versus 6–8 (*d* = 2.81, 95% CI [1.72, 3.89]); Ages 6–8 versus 9–11 (*d* = 0.84, 95% CI [0.65, 1.03]); Ages 9–11 versus 12–14 (*d* = 0.62, 95% CI [0.52, 0.71]); Ages 12–14 versus 15–17 (*d* = 0.44, 95% CI [0.27, 0.61]). All comparisons significant at *p* < .001. Heterogeneity decreased with age: *I* ^2^ = 94.24% → 94.49% → 89.23% → 33.60%, indicating more variable developmental patterns in early childhood and increasingly consistent patterns in adolescence. This hyperbolic pattern mirrors the main analysis findings, supporting the robustness of our developmental model.
**Figure S7.** Combined funnel plots excluding backward recall tasks. Egger's regression tests for funnel plot asymmetry were conducted for each age comparison: Ages 3–5 versus 6–8: *z* = 0.49, *p* = .621, indicating no significant asymmetry, though interpretation is limited by small sample size (*k* = 3). Ages 6–8 versus 9–11: *z* = 3.84, *p* < .001, indicating significant funnel plot asymmetry. Visual inspection reveals an asymmetric distribution with a deficit of small studies reporting small or negative effects, suggesting potential publication bias or small‐study effects in this age range. Ages 9–11 versus 12–14: *z* = 0.20, *p* = .844, indicating no significant asymmetry, with relatively symmetric distribution of effect sizes. Ages 12–14 versus 15–17: *z* = 1.66, *p* = .096, showing marginally non‐significant asymmetry, though interpretation is limited by small sample size (*k* = 3).
**Figure S8.** Comparison of developmental trajectories. Direct comparison of developmental trajectories from main analysis (all tasks, *k* = 316) versus sensitivity analysis (excluding backward recall tasks, *k* = 232). The solid blue line represents the main analysis trajectory; the dashed orange line represents the sensitivity analysis trajectory. Shaded regions indicate 95% confidence intervals (blue for main analysis, orange for sensitivity analysis). Grey points show observed effect sizes from the main analysis, with point size proportional to study precision (inverse variance). Both meta‐regression models demonstrate significant linear age‐related improvements in working memory updating performance across ages 3–17 years. The sensitivity analysis shows a 29% shallower developmental slope (Δ*β* = 0.05) but a 59% higher intercept (Δ*β*₀ = +0.63), indicating that backward recall tasks show steeper age‐related improvements but lower baseline performance, particularly in younger children. The trajectories converge at approximately age 11.4 years (95% CI [10.2, 12.6]), after which both predict similar performance levels.

**Data S1.** Supporting information S1.

## Data Availability

Data are available on request from the authors.
