# Supplementary material for: The developmental trajectories of working memory updating from early childhood to adolescence: A meta‐analysis
Source: Br J Psychol. 2026 Apr 5;117(4):1367–95. doi: 10.1111/bjop.70069 (PMC13655963; doi:10.1111/bjop.70069)
Supplement: Supplementary file 1 [file BJOP-117-1367-s001.pdf]

References for Analyzed Studies

Abreu-Mendoza, R. A., Chamorro, Y., Garcia-Barrera, M. A., & Matute, E. (2018). The contributions of executive functions to mathematical learning difficulties and mathematical talent during adolescence. *PloS One*, *13*(12), e0209267. https://doi.org/10.1371/journal.pone.0209267

Alesi, M., Bianco, A., Luppina, G., Palma, A., & Pepi, A. (2016). Improving Children's Coordinative Skills and Executive Functions: The Effects of a Football Exercise Program. *Perceptual and Motor Skills*, *122*(1), 27–46. https://doi.org/10.1177/0031512515627527

Andreou, M., Tsimpli, I. M., Masoura, E., & Agathopoulou, E. (2020). Cognitive Mechanisms of Monolingual and Bilingual Children in Monoliterate Educational Settings: Evidence From Sentence Repetition. *Frontiers in Psychology*, *11*, 613992. https://doi.org/10.3389/fpsyg.2020.613992

Artuso, C., Carretti, B., & Palladino, P. (2019). Short-term training on working memory updating and metacognition in primary school: The effect on reading comprehension. *School Psychology International*, *40*(6), 641–657.

Austin, G., Groppe, K., & Elsner, B. (2014). The reciprocal relationship between executive function and theory of mind in middle childhood: A 1-year longitudinal perspective. *Frontiers in Psychology*, *5*, 655. https://doi.org/10.3389/fpsyg.2014.00655

Babayiğit, S., Hitch, G. J., Kandru-Pothineni, S., Clarke, A., & Warmington, M. (2022). Vocabulary limitations undermine bilingual children's reading comprehension despite bilingual cognitive strengths. *Reading and Writing*, *35*(7), 1651–1673. https://doi.org/10.1007/s11145-021-10240-8

Basso, D., Corradini, G., & Cottini, M. (2023). "Teacher, forgive me, I forgot to do it!" The impact of children's prospective memory on teachers' evaluation of academic performance. *The British Journal of Educational Psychology*, *93*(1), 17–32. https://doi.org/10.1111/bjep.12537

Belacchi, C., Carretti, B., & Cornoldi, C. (2010). The role of working memory and updating in Coloured Raven Matrices performance in typically developing children. *European Journal of Cognitive Psychology*, *22*(7), 1010–1020.

Bisagno, E., Cadamuro, A., Rubichi, S., Robazza, C., & Vitali, F. (2022). A developmental outlook on the role of cognition and emotions in youth volleyball and artistic gymnastics. *Frontiers in Psychology*, *13*, 954820. https://doi.org/10.3389/fpsyg.2022.954820

Brandt, A., Bondü, R., & Elsner, B. (2024). Profiles of executive functions in middle childhood and prediction of later self-regulation. *Frontiers in Psychology*, *15*, 1379126. https://doi.org/10.3389/fpsyg.2024.1379126

Brydges, C. R., Fox, A. M., Reid, C. L., & Anderson, M. (2014). Predictive validity of the N2 and P3 ERP components to executive functioning in children: A latent-variable analysis. *Frontiers in Human Neuroscience*, *8*, 80. https://doi.org/10.3389/fnhum.2014.00080

Brydges, C. R., Reid, C. L., Fox, A. M., & Anderson, M. (2012). A unitary executive function predicts intelligence in children. *Intelligence*, *40*(5), 458–469. https://doi.org/10.1016/j.intell.2012.05.006

Carretti, B., Belacchi, C., & Cornoldi, C. (2010). Difficulties in working memory updating in individuals with intellectual disability. *Journal of Intellectual Disability Research*, *54*(4), 337–345. https://doi.org/10.1111/j.1365-2788.2010.01267.x

Carriedo, N., Corral, A., Montoro, P. R., Herrero, L., Ballestrino, P., & Sebastián, I. (2016). The Development of Metaphor Comprehension and Its Relationship with Relational Verbal Reasoning and Executive Function. *PloS One*, *11*(3), e0150289. https://doi.org/10.1371/journal.pone.0150289

Carriedo, N., Corral, A., Montoro, P. R., Herrero, L., & Rucián, M. (2016). Development of the updating executive function: From 7-year-olds to young adults. *Developmental Psychology*, *52*(4), 666–678. https://doi.org/10.1037/dev0000091

Chen, R., Georgiou, G. K., Peng, P., Li, Y., Li, B., Wang, J., & Tao, S. (2023). What Components of Working Memory Are Impaired in Children with Reading and/or Mathematics Difficulties? *Children (Basel, Switzerland)*, *10*(10), 1719. https://doi.org/10.3390/children10101719

Chen, X., Ye, M., Chang, L., Chen, W., & Zhou, R. (2018). Effect of Working Memory Updating Training on Retrieving Symptoms of Children With Learning Disabilities.

Journal of Learning Disabilities, *51*(5), 507–519. https://doi.org/10.1177/0022219417712015

Cheng, C., & Kibbe, M. M. (2022). Development of updating in working memory in 4–7-year-old children. *Developmental Psychology*, *58*(5), 902–912. https://doi.org/10.1037/dev0001337

Ciesielski, K. T., Lesnik, P. G., Savoy, R. L., Grant, E. P., & Ahlfors, S. P. (2006). Developmental neural networks in children performing a Categorical N-Back Task. *NeuroImage*, *33*(3), 980–990. https://doi.org/10.1016/j.neuroimage.2006.07.028

Conklin, H. M., Luciana, M., Hooper, C. J., & Yarger, R. S. (2007). Working Memory Performance in Typically Developing Children and Adolescents: Behavioral Evidence of Protracted Frontal Lobe Development. *Developmental Neuropsychology*, *31*(1), 103–128. https://doi.org/10.1207/s15326942dn3101_6

Di Lieto, M. C., Pecini, C., Castro, E., Inguaggiato, E., Cecchi, F., Dario, P., Cioni, G., & Sgandurra, G. (2019). Empowering Executive Functions in 5- and 6-Year-Old Typically Developing Children Through Educational Robotics: An RCT Study. *Frontiers in Psychology*, *10*, 3084. https://doi.org/10.3389/fpsyg.2019.03084

Diergarten, A. K., & Nieding, G. (2016). Online emotional inferences in written and auditory texts: A study with children and adults. *Reading and Writing*, *29*(7), 1383–1407. https://doi.org/10.1007/s11145-016-9642-z

Ellis Weismer, S., Kaushanskaya, M., Larson, C., Mathée, J., & Bolt, D. (2018). Executive Function Skills in School-Age Children With Autism Spectrum Disorder: Association With Language Abilities. *Journal of Speech, Language, and Hearing Research: JSLHR*, *61*(11), 2641–2658. https://doi.org/10.1044/2018_JSLHR-L-RSAUT-18-0026

Eschman, B., & Ross-Sheehy, S. (2023). Visual Short-Term Memory Persists Across Multiple Fixations: An n-Back Approach to Quantifying Capacity in Infants and Adults. *Psychological Science*, *34*(3), 370–383. https://doi.org/10.1177/09567976221136509

Estrada-Plana, V., Martínez-Escribano, A., Ros-Morente, A., Mayoral, M., Castro-Quintas, A., Vita-Barrull, N., Terés-Lleida, N., March-Llanes, J., Badia-Bafalluy, A., & Moya-Higueras, J. (2024). Benefits of Playing at School: Filler Board Games Improve

Visuospatial Memory and Mathematical Skills. *Brain Sciences*, *14*(7). https://doi.org/10.3390/brainsci14070642

Federico, F., Delogu, F., & Raffone, A. (2014). Maintenance and manipulation of object sequences in working memory: A lifespan study. *Neurological Sciences*, *35*(12), 1883–1887. https://doi.org/10.1007/s10072-014-1851-0

Finn, A. S., Minas, J. E., Leonard, J. A., Mackey, A. P., Salvatore, J., Goetz, C., West, M. R., Gabrieli, C. F. O., & Gabrieli, J. D. E. (2017). Functional brain organization of working memory in adolescents varies in relation to family income and academic achievement. *Developmental Science*, *20:12450*. https://doi.org/10.1111/desc.12450

Freis, S. M., Morrison, C. L., Lessem, J. M., Hewitt, J. K., & Friedman, N. P. (2022). Genetic and environmental influences on executive functions and intelligence in middle childhood. *Developmental Science*, *25*(1), e13150. https://doi.org/10.1111/desc.13150

Gade, M., Zoelch, C., & Seitz-Stein, K. (2017). Training of Visual-Spatial Working Memory in Preschool Children. *Advances in Cognitive Psychology*, *13*(2), 177–187. https://doi.org/10.5709/acp-0217-7

Gao, J., Li, G., Yang, Z., Li, F., Wang, T., & Wen, S. (2023). The effect of working memory updating training on the Chinese writing ability of primary school students. *Frontiers in Psychology*, *14*, 1163132. https://doi.org/10.3389/fpsyg.2023.1163132

Gao, J., Yang, Z., Li, F., Yasen, B., & Wen, S. (2023). The influence of cognitive ability in Chinese reading comprehension: Can working memory updating change Chinese primary school students' reading comprehension performance? *Frontiers in Psychology*, *14*, 1283781. https://doi.org/10.3389/fpsyg.2023.1283781

García-Madruga, J. A., Vila, J. O., Gómez-Veiga, I., Duque, G., & Elosúa, M. R. (2014). Executive processes, reading comprehension and academic achievement in 3th grade primary students. *Learning and Individual Differences*, *35*, 41–48. https://doi.org/10.1016/j.lindif.2014.07.013

Gathercole, S. E., & Pickering, S. J. (2000). Working memory deficits in children with low achievements in the national curriculum at 7 years of age. *The British Journal of Educational Psychology*, *70 (Pt 2)*, 177–194. https://doi.org/10.1348/000709900158047

Göthe, K., Esser, G., Gendt, A., & Kliegl, R. (2012). Working memory in children: Tracing age differences and special educational needs to parameters of a formal model. *Developmental Psychology*, *48*(2), 459–476. https://doi.org/10.1037/a0025660

Groppe, K., & Elsner, B. (2017). Executive function and weight status in children: A one-year longitudinal perspective. *Child Neuropsychology: A Journal on Normal and Abnormal Development in Childhood and Adolescence*, *23*(2), 129–147. https://doi.org/10.1080/09297049.2015.1089981

Hamilton, M., Roper, T., Blaser, E., & Kaldy, Z. (2024). Can't get it out of my head: Proactive interference in the visual working memory of 3- to 8-year-old children. *Developmental Psychology*, *60*(3), 582–594.

Herrero, L., & Carriedo, N. (2018). Differences in updating processes between musicians and non-musicians from late childhood to adolescence. *Learning and Individual Differences*, *61*, 188–195.

Holl, A. K., Vetter, N. C., & Elsner, B. (2021). Disentangling the relations of theory of mind, executive function and conduct problems. *Journal of Applied Developmental Psychology*, *72*, 101233. https://doi.org/10.1016/j.appdev.2020.101233

Hu, Q., Liang, Z., Zhou, Y., Feng, S., & Zhang, Q. (2023). The role of working memory updating and capacity in children's mathematical abilities: A developmental cascade model. *British Journal of Educational Psychology*, *93*(3), 676–693. https://doi.org/10.1111/bjep.12585

Im-Bolter, N., Agostino, A., & Owens-Jaffray, K. (2016). Theory of mind in middle childhood and early adolescence: Different from before? *Journal of Experimental Child Psychology*, *149*, 98–115. https://doi.org/10.1016/j.jecp.2015.12.006

Iuculano, T., Moro, R., & Butterworth, B. (2011). Updating Working Memory and arithmetical attainment in school. *Learning and Individual Differences*, *21*(6), 655–661.

Jansen, K., Tempes, J., Drozdowska, A., Gutmann, M., Falkenstein, M., Buyken, A. E., Libuda, L., Rudolf, H., Lücke, T., & Kersting, M. (2020). Short-term effects of carbohydrates differing in glycemic index (GI) consumed at lunch on children's cognitive function in a randomized crossover study. *European Journal of Clinical Nutrition*, *74*(5), 757–764. https://doi.org/10.1038/s41430-020-0600-0

Jenks, K. M., de Moor, J., & van Lieshout, E. C. D. M. (2009). Arithmetic difficulties in children with cerebral palsy are related to executive function and working memory. *Journal of Child Psychology and Psychiatry, and Allied Disciplines*, *50*(7), 824–833. https://doi.org/10.1111/j.1469-7610.2008.02031.x

Jerman, O., Reynolds, C., & Swanson, H. L. (2012). Does growth in working memory span or executive processes predict growth in reading and math in children with reading disabilities?. *Learning Disability Quarterly*, *35*(3), 144–157.

Jolles, D. D., Van Buchem, M. A., Rombouts, S. A. R. B., & Crone, E. A. (2012). Practice effects in the developing brain: A pilot study. *Developmental Cognitive Neuroscience*, *2*, S180–S191. https://doi.org/10.1016/j.dcn.2011.09.001

Karr, J. E., Rodriguez, J. E., Goh, P. K., Martel, M. M., & Rast, P. (2022). The unity and diversity of executive functions: A network approach to life span development. *Developmental Psychology*, *58*(4), 751–767. https://doi.org/10.1037/dev0001313

Kaushanskaya, M., Park, J. S., Gangopadhyay, I., Davidson, M. M., & Weismer, S. E. (2017). The Relationship Between Executive Functions and Language Abilities in Children: A Latent Variables Approach. *Journal of Speech, Language, and Hearing Research*, *60*(4), 912–923. https://doi.org/10.1044/2016_JSLHR-L-15-0310

Lechuga, M. T., Moreno, V., Pelegrina, S., Gómez-Ariza, C. J., & Bajo, M. T. (2006). Age differences in memory control: Evidence from updating and retrieval-practice tasks. *Acta Psychologica*, *123*(3), 279–298. https://doi.org/10.1016/j.actpsy.2006.01.006

Lechuga, M. T., Pelegrina, S., Pelaez, J. L., Martin-Puga, M. E., & Justicia, M. J. (2016). Working memory updating as a predictor of academic attainment. *Educational Psychology*, *36*(4), 675–690.

Lee, K., & Bull, R. (2016). Developmental changes in working memory, updating, and math achievement. *Journal of Educational Psychology*, *108*(6), 869–882.

Lee, K., Ng, S. F., Bull, R., Pe, M. L., & Ho, R. H. M. (2011). Are patterns important? An investigation of the relationships between proficiencies in patterns, computation, executive functioning, and algebraic word problems. *Journal of Educational Psychology*, *103*(2), 269–281. https://doi.org/10.1037/a0023068

Lee, K., Ng, S. F., Pe, M. L., Ang, S. Y., Hasshim, M. N. A. M., & Bull, R. (2012). The cognitive underpinnings of emerging mathematical skills: Executive functioning, patterns, numeracy, and arithmetic. *The British Journal of Educational Psychology*, *82*(Pt 1), 82–99. https://doi.org/10.1111/j.2044-8279.2010.02016.x

Lendínez, C., Pelegrina, S., & Teresa Lechuga, M. (2015). Age differences in working memory updating: The role of interference, focus switching and substituting information. *Acta Psychologica*, *157*, 106–113. https://doi.org/10.1016/j.actpsy.2015.02.015

Lensing, N., & Elsner, B. (2018). Development of hot and cool executive functions in middle childhood: Three-year growth curves of decision making and working memory updating. *Journal of Experimental Child Psychology*, *173*, 187–204. https://doi.org/10.1016/j.jecp.2018.04.002

Leopold, D. R., Kim, H., Carlson, K. W., Rowe, M. A., Groff, B. R., Major, M. P., Willcutt, E. G., Cutting, L. E., & Banich, M. T. (2024). Stimulus shapes strategy: Effects of stimulus characteristics and individual differences in academic achievement on the neural mechanisms engaged during the N-back task. *Developmental Cognitive Neuroscience*, *66*, 101372. https://doi.org/10.1016/j.dcn.2024.101372

Linares, R., Bajo, M. T., & Pelegrina, S. (2016). Age-related differences in working memory updating components. *Journal of Experimental Child Psychology*, *147*, 39–52. https://doi.org/10.1016/j.jecp.2016.02.009

Linares, R., & Pelegrina, S. (2023). The relationship between working memory updating components and reading comprehension. *Cognitive Processing*, *24*(2), 253–265. https://doi.org/10.1007/s10339-023-01127-3

Luciana, M., Conklin, H. M., Hooper, C. J., & Yarger, R. S. (2005). The development of nonverbal working memory and executive control processes in adolescents. *Child Development*, *76*(3), 697–712. https://doi.org/10.1111/j.1467-8624.2005.00872.x

Mäntylä, T., Carelli, M. G., & Forman, H. (2007). Time monitoring and executive functioning in children and adults. *Journal of Experimental Child Psychology*, *96*(1), 1–19. https://doi.org/10.1016/j.jecp.2006.08.003

Marini, A., Piccolo, B., Taverna, L., Berginc, M., & Ozbič, M. (2020). The Complex Relation between Executive Functions and Language in Preschoolers with

Developmental Language Disorders. *International Journal of Environmental Research and Public Health*, *17*(5). https://doi.org/10.3390/ijerph17051772

Massat, I., Slama, H., Kavec, M., Linotte, S., Mary, A., Baleriaux, D., Metens, T., Mendlewicz, J., & Peigneux, P. (2012). Working Memory-Related Functional Brain Patterns in Never Medicated Children with ADHD. *PLoS ONE*, *7*(11), e49392. https://doi.org/10.1371/journal.pone.0049392

Mei, S., Gao, Y., He, W., Jarrold, C., & Wang, T. (2024). Age-related differences in the removal of information from working memory. *Journal of Experimental Child Psychology*, *240*, 1–17.

Messer, D., Bernardi, M., Botting, N., Hill, E. L., Nash, G., Leonard, H. C., & Henry, L. A. (2018). An Exploration of the Factor Structure of Executive Functioning in Children. *Frontiers in Psychology*, *9*, 1179. https://doi.org/10.3389/fpsyg.2018.01179

Oppenheimer, A. V., Bellinger, D. C., Coull, B. A., Weisskopf, M. G., & Korrick, S. A. (2022). Prenatal exposure to chemical mixtures and working memory among adolescents. *Environmental Research*, *205*, 112436. https://doi.org/10.1016/j.envres.2021.112436

Palladino, P., & Ferrari, M. (2008). Phonological sensitivity and memory in children with a foreign language learning difficulty. *Memory (Hove, England)*, *16*(6), 604–625. https://doi.org/10.1080/09658210802083072

Panesi, S., Bandettini, A., Traverso, L., & Morra, S. (2022). On the Relation between the Development of Working Memory Updating and Working Memory Capacity in Preschoolers. *Journal of Intelligence*, *10*(1), 5. https://doi.org/10.3390/jintelligence10010005

Panesi, S., & Morra, S. (2022). The relation between drawing and language in preschoolers: The role of working Memory and executive functions. *Cognitive Development*, *61*, 1–20.

Papastergiou, A., Sanoudaki, E., Tamburelli, M., & Chondrogianni, V. (2023). A study on the executive functioning skills of Greek–English bilingual children – a nearest neighbour approach. *Bilingualism: Language and Cognition*, *26*(1), 78–94. https://doi.org/10.1017/s1366728922000335

Passolunghi, M. C., & Cornoldi, C. (2008). Working Memory Failures in Children with Arithmetical Difficulties. *Child Neuropsychology*, *14*(5), 387–400. https://doi.org/10.1080/09297040701566662

Passolunghi, M. C., De Blas, G. D., Carretti, B., Gomez-Veiga, I., Doz, E., & Garcia-Madruga, J. A. (2022). The role of working memory updating, inhibition, fluid intelligence, and reading comprehension in explaining differences between consistent and inconsistent arithmetic word-problem-solving performance. *Journal of Experimental Child Psychology*, *224*, 105512. https://doi.org/10.1016/j.jecp.2022.105512

Pelegrina, S., Capodieci, A., Carretti, B., & Cornoldi, C. (2015). Magnitude Representation and Working Memory Updating in Children With Arithmetic and Reading Comprehension Disabilities. *Journal of Learning Disabilities*, *48*(6), 658–668. https://doi.org/10.1177/0022219414527480

Pelegrina, S., Lechuga, M. T., García-Madruga, J. A., Elosúa, M. R., Macizo, P., Carreiras, M., Fuentes, L. J., & Bajo, M. T. (2015). Normative data on the n-back task for children and young adolescents. *Frontiers in Psychology*, *6*. https://search.ebscohost.com/login.aspx?direct=true&db=psyh&AN=2016-22593-001

Pelegrina, S., Molina, R., Rodríguez-Martínez, E. I., Linares, R., & Gómez, C. M. (2020). Age-related changes in selection, recognition, updating and maintenance information in WM. An ERP study in children and adolescents. *Biol Psychol*, *157*, 107977. https://doi.org/10.1016/j.biopsycho.2020.107977

Peng, P., Congying, S., Beilei, L., & Sha, T. (2012). Phonological storage and executive function deficits in children with mathematics difficulties. *Journal of Experimental Child Psychology*, *112*(4), 452–466. https://doi.org/10.1016/j.jecp.2012.04.004

Peng, P., Sha, T., & Li, B. (2013). The deficit profile of working memory, inhibition, and updating in Chinese children with reading difficulties. *Learning and Individual Differences*, *25*, 111–117.

Rohlf, H. L., Holl, A. K., Kirsch, F., Krahé, B., & Elsner, B. (2018). Longitudinal Links between Executive Function, Anger, and Aggression in Middle Childhood. *Frontiers in Behavioral Neuroscience*, *12*, 27. https://doi.org/10.3389/fnbeh.2018.00027

Rose, S. A., Feldman, J. F., & Jankowski, J. J. (2011). Modeling a cascade of effects: The role of speed and executive functioning in preterm/full-term differences in academic achievement. *Developmental Science*, *14*(5), 1161–1175. https://doi.org/10.1111/j.1467-7687.2011.01068.x

Salas, N., & Silvente, S. (2020). The role of executive functions and transcription skills in writing: A cross-sectional study across 7 years of schooling. *Reading and Writing*, *33*(4), 877–905. https://doi.org/10.1007/s11145-019-09979-y

Santos, F. H., & Bueno, O. F. A. (2003). Validation of the Brazilian Children's Test of Pseudoword Repetition in Portuguese speakers aged 4 to 10 years. *Brazilian Journal of Medical and Biological Research = Revista Brasileira De Pesquisas Medicas E Biologicas*, *36*(11), 1533–1547. https://doi.org/10.1590/s0100-879x2003001100012

St Clair-Thompson, H. L., & Gathercole, S. E. (2006). Executive functions and achievements in school: Shifting, updating, inhibition, and working memory. *Quarterly Journal of Experimental Psychology*, *59*(4), 745–759. https://doi.org/10.1080/17470210500162854

Swanson, H. L., Howard, C. B., & Sáez, L. (2006). Do different components of working memory underlie different subgroups of reading disabilities? *Journal of Learning Disabilities*, *39*(3), 252–269. https://doi.org/10.1177/00222194060390030501

Traverso, L., Viterbori, P., Malagoli, C., & Usai, M. C. (2020). Distinct inhibition dimensions differentially account for working memory performance in 5-year-old children. *Cognitive Development*, *55*. https://search.ebscohost.com/login.aspx?direct=true&db=psyh&AN=2020-62122-001

Van Der Sluis, S., De Jong, P. F., & Van Der Leij, A. (2007). Executive functioning in children, and its relations with reasoning, reading, and arithmetic. *Intelligence*, *35*(5), 427–449. https://doi.org/10.1016/j.intell.2006.09.001

Van Der Ven, S. H. G., Kroesbergen, E. H., Boom, J., & Leseman, P. P. M. (2012). The development of executive functions and early mathematics: A dynamic relationship. *British Journal of Educational Psychology*, *82*(1), 100–119. https://doi.org/10.1111/j.2044-8279.2011.02035.x

Visu-Petra, L., Cheie, L., Benga, O., & Packiam Alloway, T. (2011). Effects of anxiety on memory storage and updating in young children. *International Journal of Behavioral Development*, *35*(1), 38–47.

Visu-Petra, L., Ţincaş, I., Cheie, L., & Benga, O. (2010). Anxiety and visual-spatial memory updating in young children: An investigation using emotional facial expressions. *Cognition and Emotion*, *24*(2), 223–240.

Völter, C. J., Reindl, E., Felsche, E., Civelek, Z., Whalen, A., Lugosi, Z., Duncan, L., Herrmann, E., Call, J., & Seed, A. M. (2022). The structure of executive functions in preschool children and chimpanzees. *Scientific Reports*, *12*(1), 6456. https://doi.org/10.1038/s41598-022-08406-7

Whitely, C., & Colozzo, P. (2013). Who's Who? Memory Updating and Character Reference in Children's Narratives. *Journal of Speech, Language, and Hearing Research*, *56*(5), 1625–1636. https://doi.org/10.1044/1092-4388(2013/12-0176)

Wood, A. C., Vainik, U., Engelhardt, L. E., Briley, D. A., Grotzinger, A. D., Church, J. A., Harden, K. P., & Tucker-Drob, E. M. (2019). Genetic overlap between executive functions and BMI in childhood. *The American Journal of Clinical Nutrition*, *110*(4), 814–822. https://doi.org/10.1093/ajcn/nqz109

Xu, F., Han, Y., Sabbagh, M. A., Wang, T., Ren, X., & Li, C. (2013). Developmental Differences in the Structure of Executive Function in Middle Childhood and Adolescence. *PLoS ONE*, *8*(10), e77770. https://doi.org/10.1371/journal.pone.0077770

Yen, M.-H., Han, C.-C., Yu, P.-C., Yang, T.-H., Didino, D., Butterworth, B., & Yen, N.-S. (2017). The influence of memory updating and number sense on junior high school math attainment. *Learning and Individual Differences*, *54*, 30–40.

Young, E. S., Frankenhuis, W. E., DelPriore, D. J., & Ellis, B. J. (2022). Hidden talents in context: Cognitive performance with abstract versus ecological stimuli among adversity-exposed youth. *Child Development*, *93*(5), 1493–1510. https://doi.org/10.1111/cdev.13766

Zhang, H., Chang, L., Chen, X., Ma, L., & Zhou, R. (2018). Working Memory Updating Training Improves Mathematics Performance in Middle School Students With Learning

Difficulties. *Frontiers in Human Neuroscience*, *12*, 154. https://doi.org/10.3389/fnhum.2018.00154

Zhao, X., Cao, W., & Maes, J. H. R. (2023). Association between adolescents' socioeconomic status and working memory updating functioning: Role of parental educational involvement. *Journal of Research on Adolescence*, *33*(3), 931–942.

Zhao, X., Fu, J., Ma, X., & Maes, J. H. R. (2019). Age Differences in Prospective Memory: A Further Evaluation of the Executive Framework. *Journal of Cognition and Development*, *20*(5), 680–701. https://doi.org/10.1080/15248372.2019.1648268

Zhao, X., & Jia, L. (2019). Training and transfer effects of interference control training in children and young adults. *Psychological Research*, *83*(7), 1519–1530. https://doi.org/10.1007/s00426-018-1007-6

Zhao, X., Wang, Y., & Maes, J. H. R. (2022). The effect of working memory capacity and training on intertemporal decision making in children from low-socioeconomic-status families. *Journal of Experimental Child Psychology*, *216*, 105347. https://doi.org/10.1016/j.jecp.2021.105347

Zhao, X., Zhang, W., Tong, D., & Maes, J. H. R. (2023). Creative thinking and executive functions: Associations and training effects in adolescents. *Psychology of Aesthetics, Creativity, and the Arts*, *17*(1), 79–90. https://doi.org/10.1037/aca0000392

Zhou, Y., Yu, N. X., Dong, P., & Zhang, Q. (2022). Stressful life events and children's socioemotional difficulties: Conditional indirect effects of resilience and executive function. *Journal of Experimental Child Psychology*, *216*, 105345. https://doi.org/10.1016/j.jecp.2021.105345
