# Supplementary material for: The developmental trajectories of working memory updating from early childhood to adolescence: A meta‐analysis
Source: Br J Psychol. 2026 Apr 5;117(4):1367–95. doi: 10.1111/bjop.70069 (PMC13655963; doi:10.1111/bjop.70069)
Supplement: Supplementary file 2 [file BJOP-117-1367-s002.pdf]

Supplementary Materials

Table S1

Quality Index Criteria and Scoring

Participant Characteristics	0	1	2
Age data reported?	Not reported	Partially reported	Fully reported
Gender reported?	Not reported	Reported	NA
Recruitment process reported?	Not reported	Partially reported	Fully reported

Task Procedure	0	1	2
Familiarization trail included?	Not reported	Trail Included	NA
Task validated?	Not validated	Partially validated	Validated measure
Task parameters justified?	Not justified	Partially justified	Fully justified
Administration standardized?	Not standardized	Partially standardized	Fully standardized
Testing environment controlled?	Not reported	Partially controlled	Fully Controlled
Outcome measures defined?	Not defined	Partially defined	Fully defined and justified

Note. Quality assessment criteria with scoring range 0-2 for each dimension. Maximum possible score = 16. NA = Not Applicable.

Table S2

Scores for individual studies included in the analysis

Study	Age data reported?	Gender reported?	Recruitment process reported?	Familiarization trail included?	Task validated?	Task parameters justified?	Administration standardized?	Testing environment controlled?	Outcome measures defined?	Total Score
Abreu-Mendoza et al., 2018	2	1	2	0	1	2	2	2	2	14
Alesi et al., 2016	2	1	1	0	1	2	1	1	2	11
Andreou et al., 2020	2	1	2	1	1	2	1	1	2	13
Artuso et al., 2019	2	1	1	1	1	2	2	2	2	14
Austin et al., 2014	2	1	2	0	2	0	2	1	2	12
Babayiğit et al., 2022	2	0	1	0	2	2	2	1	2	12
Basso et al., 2023	2	1	2	1	2	1	2	1	2	14
Belacchi et al., 2010	2	1	0	1	1	0	2	1	2	10
Bisagno et al., 2022	2	1	1	0	2	2	2	2	2	14
Brandt et al., 2024	2	1	2	1	2	2	2	2	2	16
Brydges et al., 2014	2	0	0	0	2	2	2	2	2	12
Brydges et al., 2012	2	0	0	0	2	2	1	1	2	10
Carretti et al., 2010	2	1	1	1	2	2	2	1	2	14
Carriedo et al., 2016a	2	1	1	1	2	1	2	2	2	14
Carriedo et al., 2016b	2	1	1	1	2	2	2	2	2	15
Chen et al., 2023	2	0	1	0	2	2	1	1	2	11
Chen et al., 2018	2	1	2	1	2	2	2	1	2	15
Cheng & Kibbe., 2022	2	1	2	1	2	2	2	1	2	15
Ciesielski et al., 2006	2	0	0	0	2	2	2	2	2	12
Conklin et al., 2007	2	1	2	1	2	2	2	2	2	16
Di Lieto et al., 2019	2	1	2	1	2	2	2	1	2	15
Diergarten & Nieding, 2016	2	1	0	0	2	2	2	1	2	12
Ellis Weismer et al., 2018	2	1	1	1	2	1	2	2	2	14
Eschman & Ross-Sheehy., 2023	2	1	2	1	2	2	2	2	2	16
Estrada-Plana et al., 2024	2	0	2	0	2	2	2	2	2	14
Federico et al., 2014	2	0	0	0	2	2	1	1	2	10
Finn et al., 2017	1	1	1	0	2	2	2	2	2	13
Freis et al., 2022	1	1	2	0	2	2	2	2	2	14
Gade et al., 2017	2	1	1	1	2	1	2	2	2	14

Gao et al., 2023a	2	1	2	2	2	1	2	2	2	16
Gao et al., 2023b	2	1	2	1	2	0	2	2	2	14
García-Madruga et al., 2014	1	1	0	0	2	2	1	1	2	10
Gathercole & Pickering., 2000	2	1	1	1	2	1	2	2	2	14
Göthe et al., 2012	2	0	2	1	2	2	2	2	2	15
Groppe & Elsner., 2017	2	1	2	1	2	2	2	2	2	16
Hamilton et al., 2024	2	1	1	1	2	2	2	1	2	14
Herrero & Carriedo., 2019	2	1	1	1	2	2	2	1	2	14
Holl et al., 2021	2	0	1	0	2	2	2	1	2	12
Hu et al., 2023	2	1	1	0	2	1	2	1	2	12
Iuculano et al., 2011	1	0	2	1	2	2	2	1	2	13
Jansen et al., 2020	2	1	2	1	2	1	2	1	2	14
Jenks et al., 2009	2	1	2	1	1	1	2	2	2	14
Jerman et al., 2012	2	1	2	0	2	2	2	1	2	14
Jolles et al., 2012	2	1	2	1	2	2	2	2	2	16
Karr et al., 2022	2	1	1	1	2	1	1	1	1	11
Kaushanskaya et al., 2017	2	1	2	0	2	2	2	2	2	15
Lechuga et al., 2006	2	1	1	1	2	2	2	1	2	14
Lechuga et al., 2016	1	1	0	0	2	2	1	1	2	10
Lee & Bull., 2016	2	1	2	1	2	2	2	1	2	15
Lee et al., 2011	2	0	0	0	2	2	2	1	2	11
Lee et al., 2012	2	0	1	0	2	2	2	1	2	12
Lensing & Elsner., 2018	2	1	2	1	2	2	2	2	2	14
Lendínez et al., 2015	2	1	1	1	2	2	2	1	2	16
Leopold et al., 2024	2	1	1	0	2	2	2	2	2	14
Linares et al., 2016	2	1	2	1	2	2	2	2	2	16
Linares & Pelegrina., 2023	2	1	1	1	2	2	1	1	2	13
Luciana et al., 2005	2	1	0	0	2	2	1	1	2	11
Mäntylä et al., 2007	2	1	2	1	2	2	2	2	2	16
Massat et al., 2012	2	1	1	1	2	1	2	2	2	14
Mei et al., 2024	2	0	2	1	2	2	2	2	2	15
Messer et al., 2018	2	1	2	0	2	1	2	1	2	13
Oppenheimer et al., 2022	2	1	2	0	2	2	1	1	2	13
Palladino & Ferrari., 2008	2	1	1	1	2	2	2	1	2	14
Panesi et al., 2022	2	1	2	0	2	0	2	2	2	13
Panesi & Morra, 2022	2	1	2	0	2	2	2	1	2	14
Passolunghi & Cornoldi., 2008	1	1	2	1	2	1	2	1	2	13
Passolunghi et al., 2022	1	1	1	1	2	2	1	1	2	12
Pelegrina et al., 2015	2	1	2	1	2	2	2	1	2	15
Pelegrina et al., 2020	2	1	2	1	2	2	2	2	2	16

Peng et al., 2012	1	1	0	0	2	2	1	1	2	10
Peng et al., 2013	1	1	2	1	1	0	1	1	2	10
Rohlf et al., 2018	2	1	2	1	2	1	2	2	2	15
Rose et al., 2011	2	1	2	1	2	2	2	2	2	16
Salas & Bueno, 2020	2	1	0	0	2	2	1	1	2	11
Santos & Bueno., 2003	2	1	2	1	2	2	1	1	2	14
St Clair-Thompson & Gathercole., 2006	2	1	1	1	2	2	2	1	2	14
Swanson et al., 2006	2	1	1	1	2	1	2	2	2	14
Traverso et al., 2020	2	1	2	1	2	1	2	1	2	14
Van Der Sluis et al., 2007	1	1	0	0	2	2	2	1	2	11
Van Der Ven et al., 2012	2	1	1	0	2	2	2	2	2	14
Visu-Petra et al., 2011	1	1	0	0	2	2	1	1	2	10
Visu-Petra et al., 2010	2	1	2	1	2	2	2	1	2	15
Völter et al., 2022	2	1	2	1	2	1	2	2	2	15
Whitely & Colozzo., 2013	2	1	2	1	2	2	2	2	2	16
Wood et al., 2019	2	1	2	0	2	1	2	2	2	14
Xu et al., 2013	2	1	1	1	2	2	2	1	2	14
Yen et al., 2017	2	1	1	1	2	1	2	1	2	13
Young et al., 2022	2	1	2	0	2	2	2	1	2	14
Zhang et al., 2018	2	1	1	1	2	1	2	1	2	13
Zhao et al., 2019	2	1	1	1	2	1	2	1	2	13
Zhao et al., 2023	2	1	2	1	2	1	2	1	2	14
Zhao et al., 2019	2	1	1	1	2	1	2	2	2	14
Zhao et al., 2022	2	1	2	1	2	2	2	1	2	15
Zhao et al., 2023	2	1	1	0	2	2	2	2	2	14
Zhou et al., 2022	2	1	1	0	2	2	2	1	2	13

Note. Individual quality scores for each study based on criteria in Table 1 (0 = not reported/not standardized, 1 = partially reported/standardized, 2 = fully reported/standardized). Total Score = sum of all criteria (maximum = 16).

Figure S1

Forest Plot for Comparison between Age Groups 3-5 and 6-8 years.

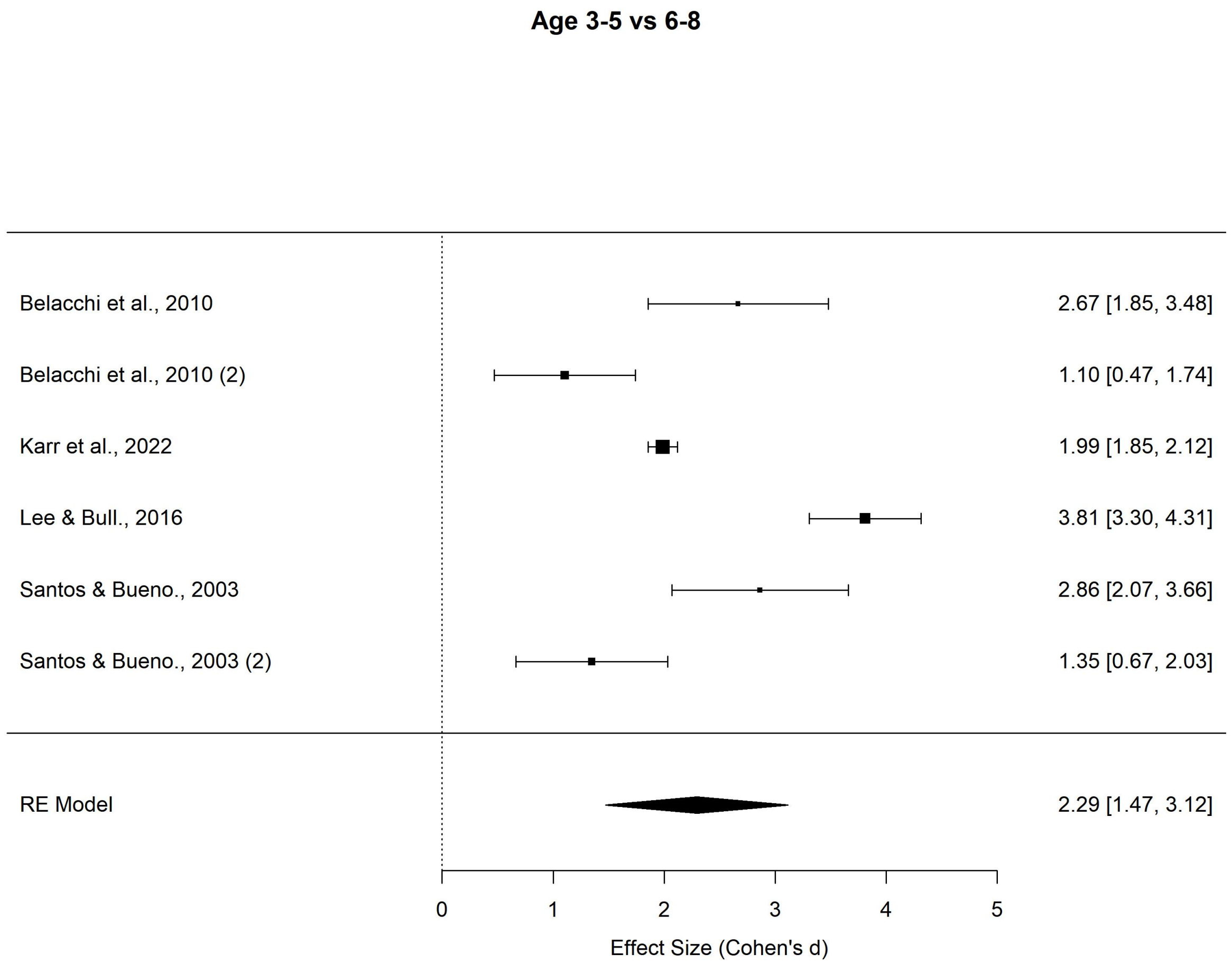

Note. Forest plot showing effect sizes (Cohen's d) and 95% confidence intervals for individual studies comparing working memory performance between younger (3-5 years) and older (6-8 years) children. The diamond represents the pooled random-effects estimate (d = 2.29, 95% CI [1.47, 3.12]). Positive values indicate better performance in the older age group.

Figure S2

Forest Plot for Comparison between Age Groups 6-8 and 9-11 years.

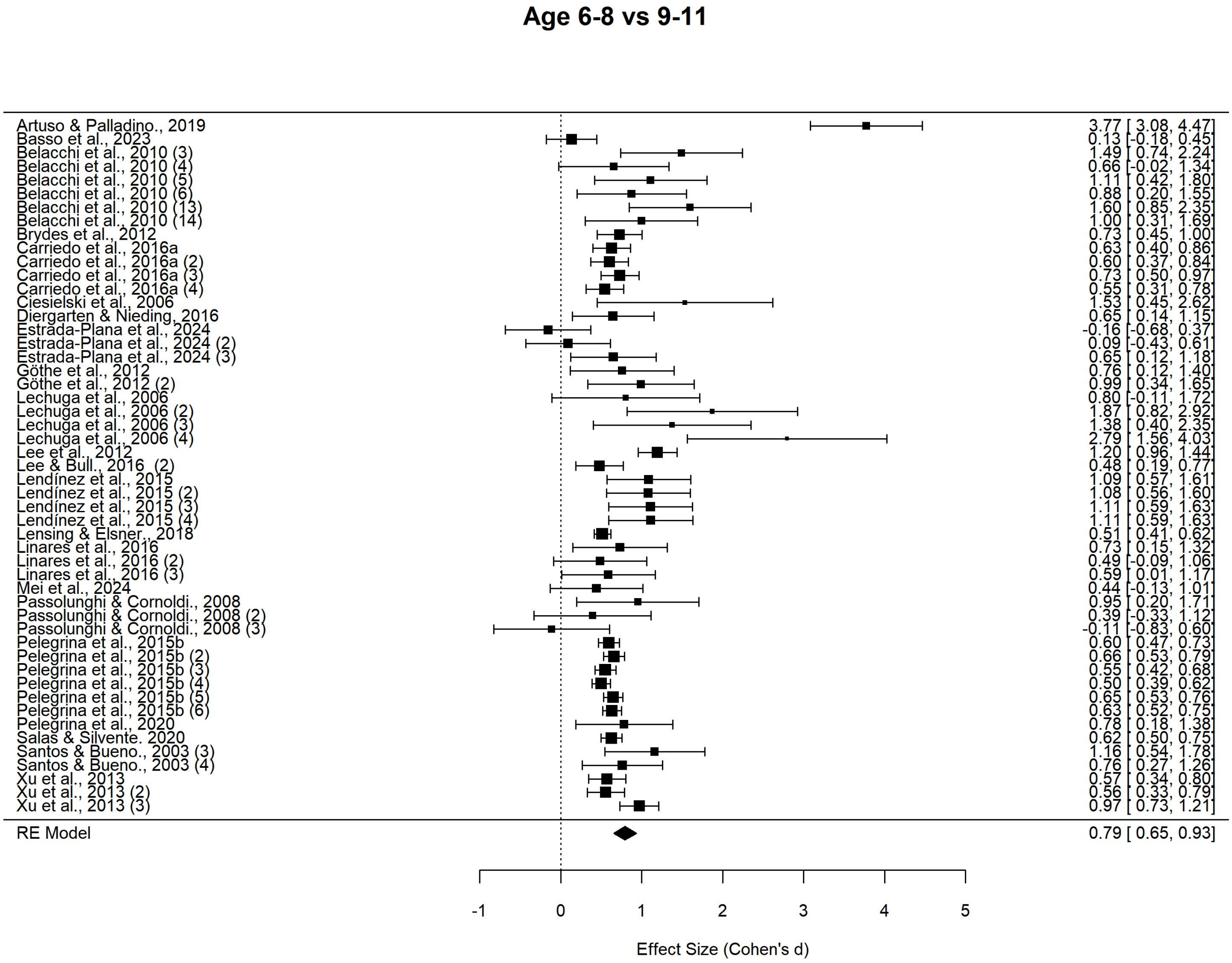

Note. Forest plot comparing working memory performance between age groups 6-8 and 9-11 years. Pooled random-effects estimate: d = 0.79, 95% CI [0.65, 0.93].

Figure S3

Forest Plot for Comparison between Age Groups 9-11 and 12-14 years.

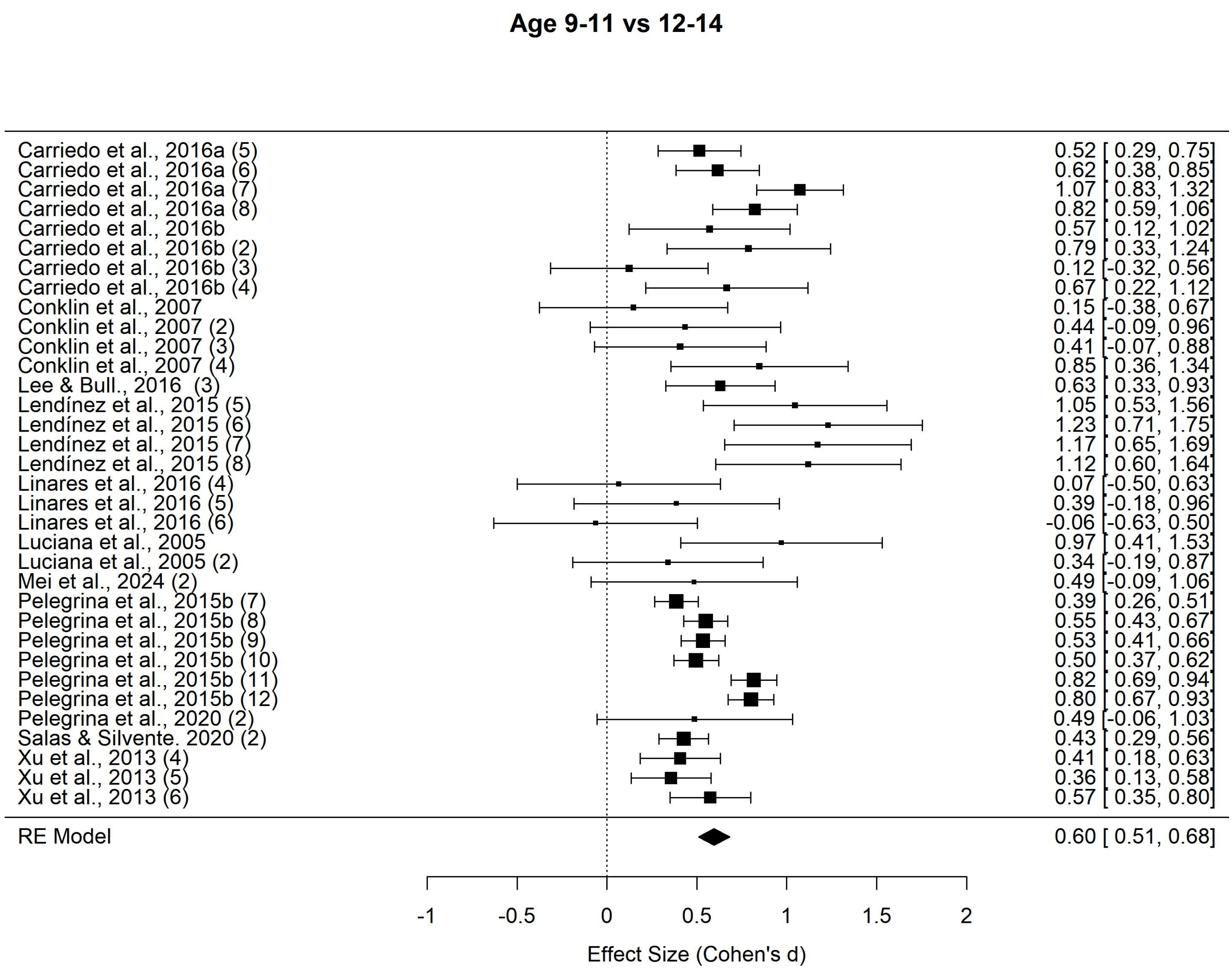

Note. Forest plot comparing working memory performance between age groups 9-11 and 12-14 years. Pooled random-effects estimate: d = 0.60, 95% CI [0.51, 0.68].

Figure S4

Forest Plot for Comparison between Age Groups 12-14 and 15-17 years.

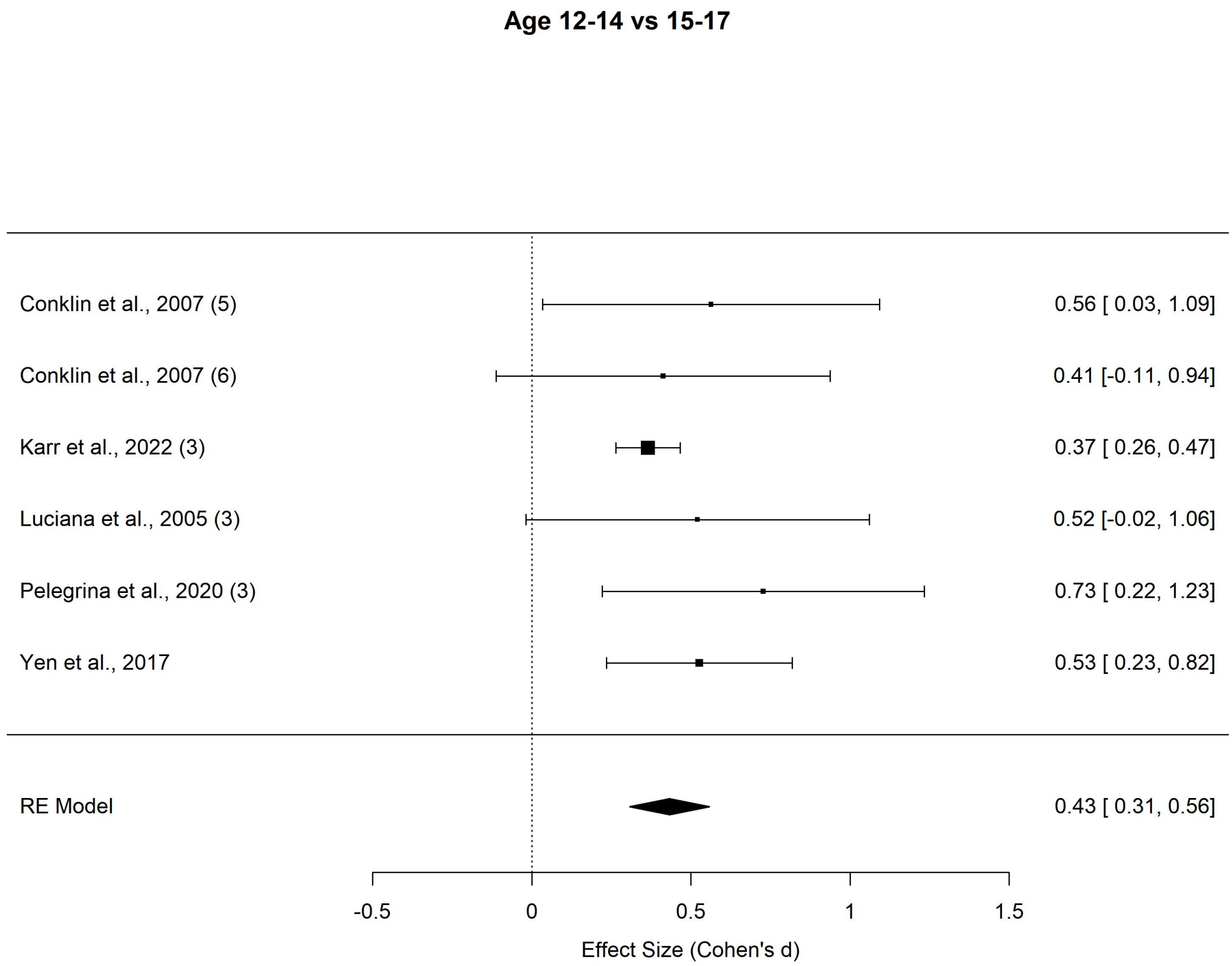

Note. Forest plot comparing working memory performance between age groups 12-14 and 15-17 years. Pooled random-effects estimate: d = 0.43, 95% CI [0.31, 0.56].

Figure S5

Developmental Trajectory Excluding Backward Recall Tasks.

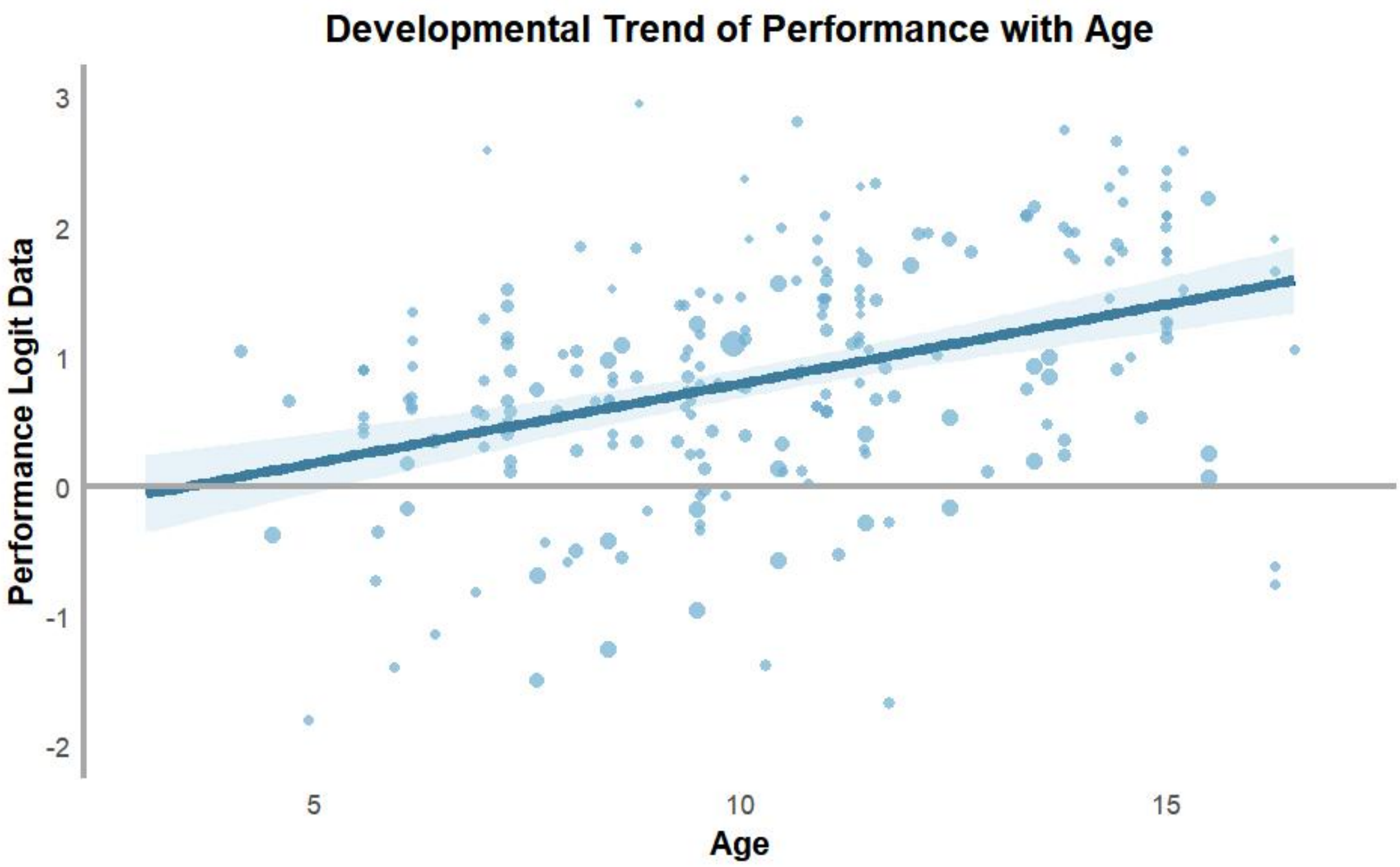

Note. This sensitivity analysis excludes all backward recall tasks. The meta-regression model showed significant age-related improvement ($\beta = 0.122$, $SE = 0.019$, $p < .001$), with age accounting for 17.38% of variance in performance ($R^2 = 17.38\%$). Substantial heterogeneity remained after accounting for age ($I^2 = 94.64\%$, $\tau^2 = 0.60$).

Figure S6

Combined Forest Plots Excluding Backward Recall Tasks.

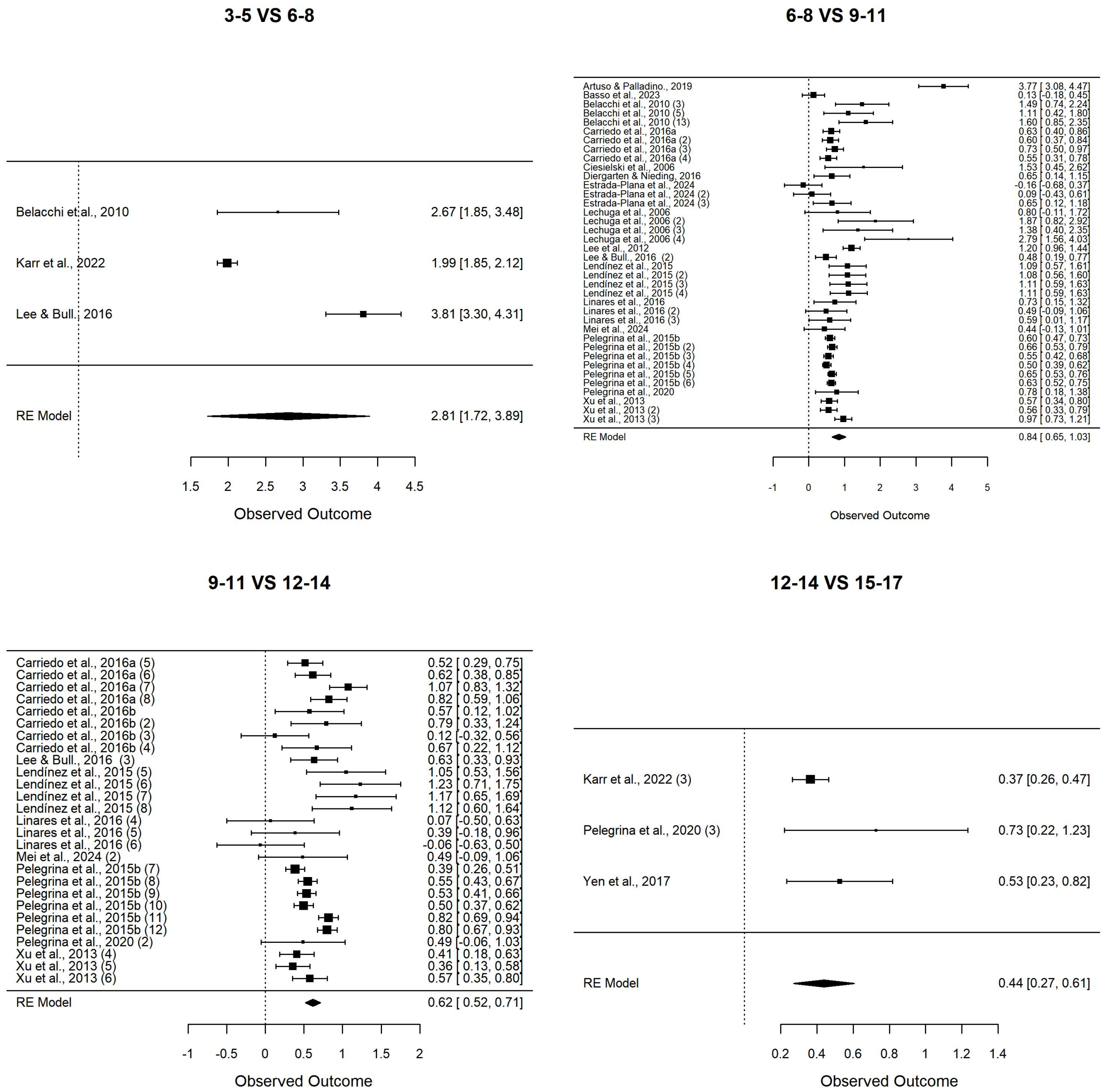

Note. Top left panel: Ages 3-5 vs. 6-8 (k = 3); Top right panel: Ages 6-8 vs. 9-11 (k = 38); Bottom left panel: Ages 9-11 vs. 12-14 (k = 31); Bottom right panel: Ages 12-14 vs. 15-17 (k = 3). Random-effects meta-analyses across four age comparisons, excluding backward recall tasks (total k = 75 effect sizes). Effect sizes show systematic developmental deceleration: Ages 3-5 vs. 6-8 (d = 2.81, 95% CI [1.72, 3.89]); Ages 6-8 vs. 9-11 (d = 0.84, 95% CI [0.65, 1.03]); Ages 9-11 vs. 12-14 (d = 0.62, 95% CI [0.52, 0.71]); Ages 12-14 vs. 15-17 (d = 0.44, 95% CI [0.27, 0.61]). All comparisons significant at $p < .001$. Heterogeneity decreased with age: I^2 = 94.24% → 94.49% → 89.23% → 33.60%, indicating more variable developmental patterns in early childhood and increasingly consistent patterns in adolescence. This hyperbolic pattern mirrors the main analysis findings, supporting the robustness of our developmental model.

Figure S7

Combined Funnel Plots Excluding Backward Recall Tasks.

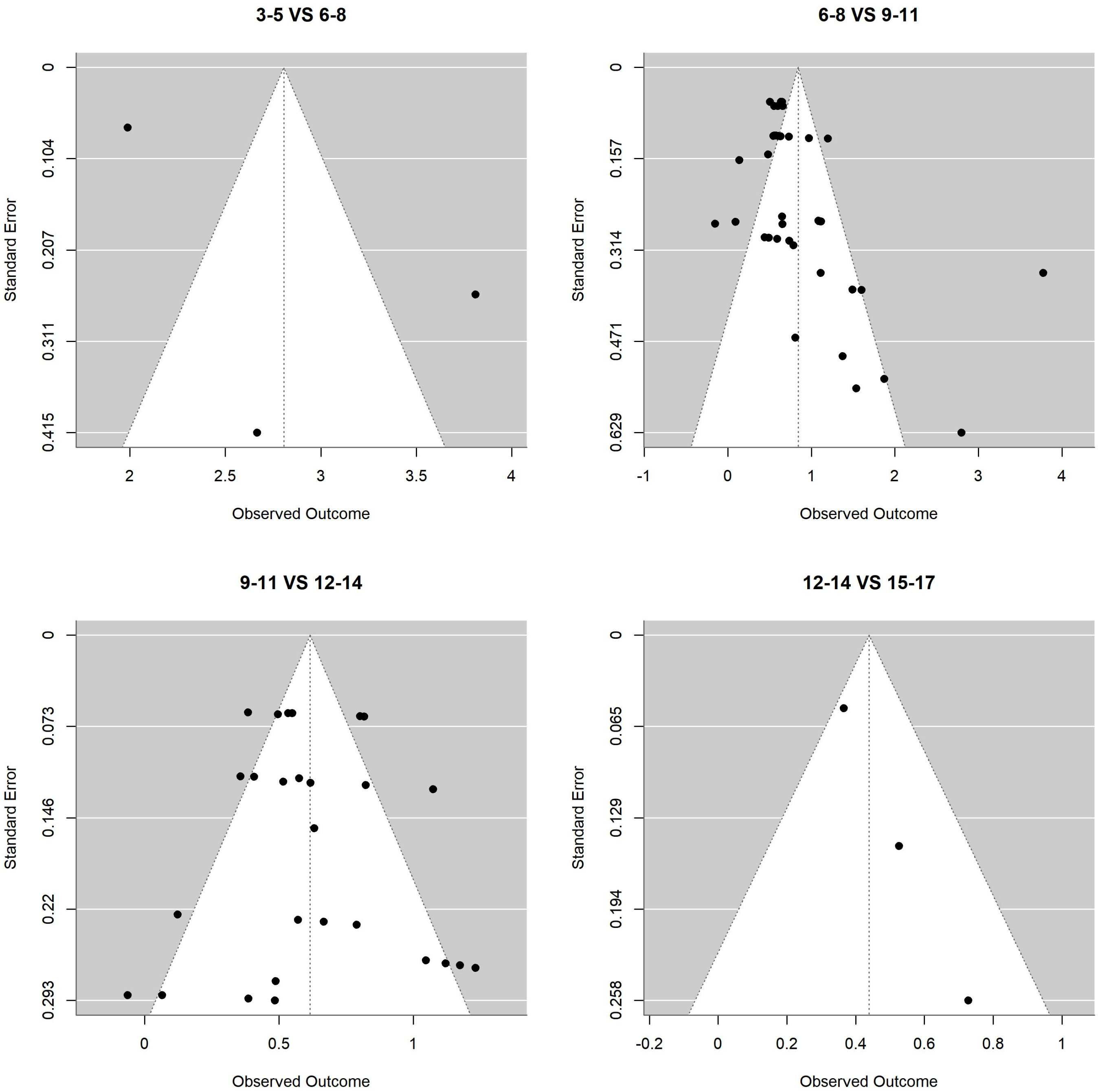

Note. Egger's regression tests for funnel plot asymmetry were conducted for each age comparison: Ages 3-5 vs. 6-8: $z = 0.49$, $p = .621$, indicating no significant asymmetry, though interpretation is limited by small sample size ($k = 3$). Ages 6-8 vs. 9-11: $z = 3.84$, $p < .001$, indicating significant funnel plot asymmetry. Visual inspection reveals an asymmetric distribution with a deficit of small studies reporting small or negative effects, suggesting potential publication bias or small-study effects in this age range. Ages 9-11 vs. 12-14: $z = 0.20$, $p = .844$, indicating no significant asymmetry, with relatively symmetric distribution of effect sizes. Ages 12-14 vs. 15-17: $z = 1.66$, $p = .096$, showing marginally non-significant asymmetry, though interpretation is limited by small sample size ($k = 3$).

Figure S8

Comparison of Developmental Trajectories.

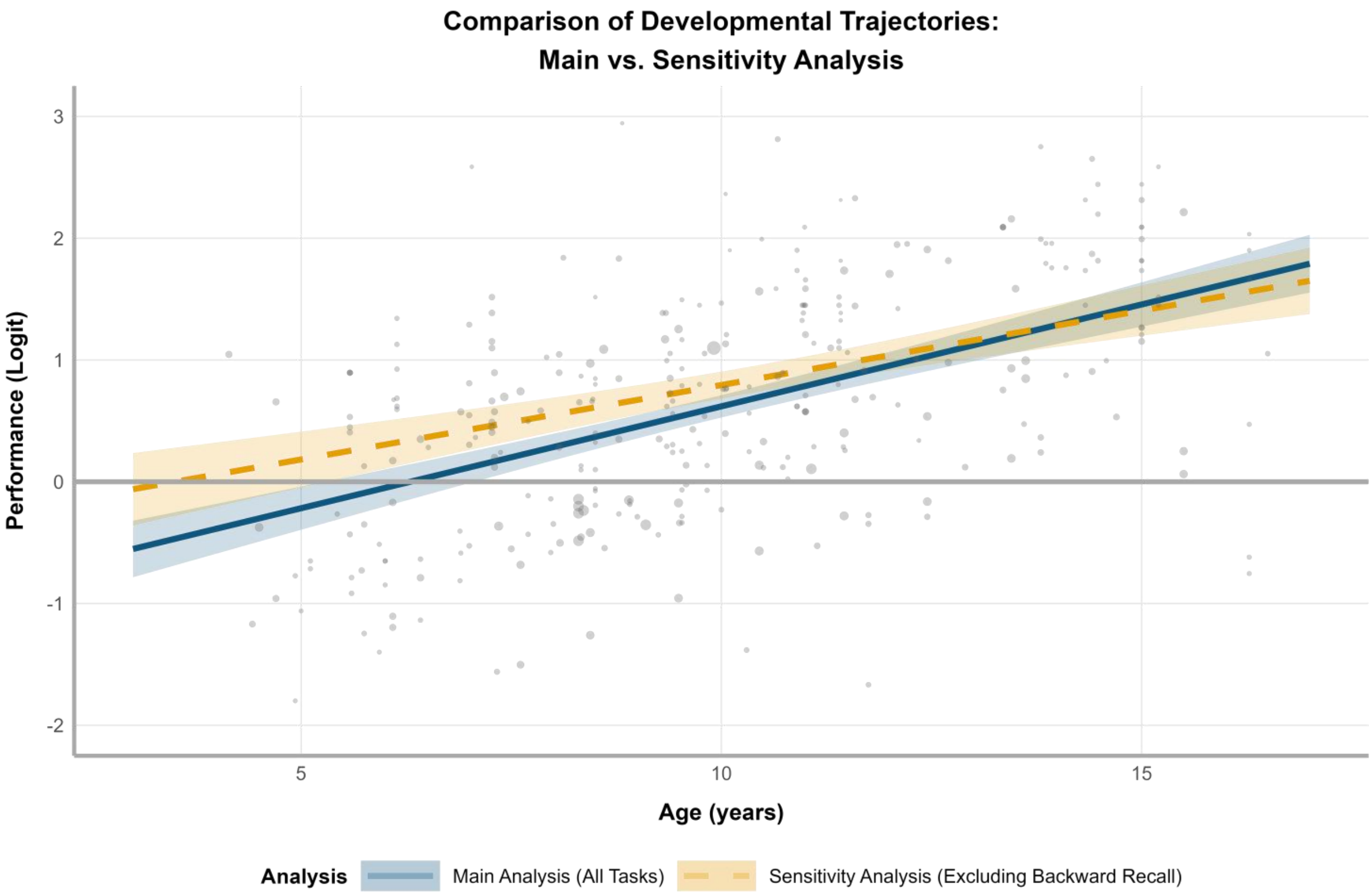

Note. Direct comparison of developmental trajectories from main analysis (all tasks, k = 316) versus sensitivity analysis (excluding backward recall tasks, k = 232). The solid blue line represents the main analysis trajectory; the dashed orange line represents the sensitivity analysis trajectory. Shaded regions indicate 95% confidence intervals (blue for main analysis, orange for sensitivity analysis). Gray points show observed effect sizes from the main analysis, with point size proportional to study precision (inverse variance). Both meta-regression models demonstrate significant linear age-related improvements in working memory updating performance across ages 3-17 years. The sensitivity analysis shows a 29% shallower developmental slope ($\Delta\beta$ = 0.05) but a 59% higher intercept ($\Delta\beta_0$ = +0.63), indicating that backward recall tasks show steeper age-related improvements but lower baseline performance, particularly in younger children. The trajectories converge at approximately age 11.4 years (95% CI [10.2, 12.6]), after which both predict similar performance levels.
